# Akt is a mediator of artery specification during zebrafish development

**DOI:** 10.1242/dev.202727

**Published:** 2024-09-02

**Authors:** Wenping Zhou, Joey J. Ghersi, Emma Ristori, Nicole Semanchik, Andrew Prendergast, Rong Zhang, Paola Carneiro, Gabriel Baldissera, William C. Sessa, Stefania Nicoli

**Affiliations:** ^1^ Vascular Biology & Therapeutics Program, Yale University School of Medicine, New Haven, CT 06520, USA; ^2^ Department of Cell Biology, Yale University School of Medicine, New Haven, CT 06511, USA; ^3^ Department of Pharmacology, Yale University School of Medicine, New Haven, CT 06510, USA; ^4^ Yale Cardiovascular Research Center, Department of Internal Medicine, Section of Cardiology, Yale University School of Medicine, New Haven, CT 06511, USA; ^5^ Department of Genetics, Yale University School of Medicine, New Haven, CT 06510, USA; ^6^ Pathologies Foetomaternelles et Néonatales, Centre Hospitalier Universitaire Sainte-Justine Research Center, Montréal, QC H3T 1C5, Canada; ^7^ Department of Pathology and Cell Biology, Faculty of Medicine, Université de Montréal, Montréal, QC H3T 1J4, Canada; ^8^ Department of Comparative Medicine, Yale zebrafish Research Core, Yale University School of Medicine, New Haven, CT 06510, USA

**Keywords:** Zebrafish embryo, Vascular development, Endothelial cells, Artery specification, Akt and Notch signaling, Single cell RNA sequencing

## Abstract

The dorsal aorta (DA) is the first major blood vessel to develop in the embryonic cardiovascular system. Its formation is governed by a coordinated process involving the migration, specification, and arrangement of angioblasts into arterial and venous lineages, a process conserved across species. Although vascular endothelial growth factor a (VEGF-A) is known to drive DA specification and formation, the kinases involved in this process remain ambiguous. Thus, we investigated the role of protein kinase B (Akt) in zebrafish by generating a quadruple mutant (*akt*^Δ/Δ^), in which expression and activity of all Akt genes – *akt1*, *-2*, *-3a* and *-3b* – are strongly decreased. Live imaging of developing *akt*^Δ/Δ^ DA uncovers early arteriovenous malformations. Single-cell RNA-sequencing analysis of *akt*^Δ/Δ^ endothelial cells corroborates the impairment of arterial, yet not venous, cell specification. Notably, endothelial specific expression of ligand-independent activation of Notch or constitutively active Akt1 were sufficient to re-establish normal arterial specification in *akt*^Δ/Δ^. The Akt loss-of-function mutant unveils that Akt kinase can act upstream of Notch in arterial endothelial cells, and is involved in proper embryonic artery specification. This sheds light on cardiovascular development, revealing a mechanism behind congenital malformations.

## Teaser

**Highlighted Article:** The first complete loss-of-function mutant in zebrafish involving multiple Akt alleles highlights the necessity of Akt signaling in sustaining the arterial specification and formation of the dorsal aorta through the maintenance of the Notch pathway.

## INTRODUCTION

The cardiovascular system plays a crucial role in supplying all tissues and organs with oxygen and nutrients. The development of all vertebrates is dependent on proper formation of this system (Majesky, 2018). The dorsal aorta (DA) is the first blood vessel that forms during embryogenesis, followed by the posterior cardinal vein (PCV). These vessels are derived from angioblasts (endothelial progenitors) originating in the posterior lateral plate mesoderm. They then migrate to the embryo midline, where they are specified to differentiate into artery or vein endothelial cells (Herbert et al., 2009; Lawson and Weinstein, 2002a). With the formation of the vascular lumen and the initiation of blood circulation, the DA and PCV acquire functional differences in vascular diameter, extracellular matrix and smooth muscle cell coverage that are maintained into adulthood (dela Paz and D'Amore, 2009; Lawson and Weinstein, 2002a; Trimm and Red-Horse, 2023). Importantly, the molecular programs that dictate the development of the DA and PCV are conserved across vertebrate models (Gore et al., 2012; Isogai et al., 2001).

Arterial and venous specification are genetically programmed during development and involve the hierarchical organization of multiple signaling pathways (Chappell and Bautch, 2010). Artery specification is orchestrated by two powerful arteriogenic signaling pathways, the Vascular endothelial growth factor a (Vegfa) and the Notch pathways (Chen et al., 2021). Vegfa and Notch signaling are highly interdependent in vascular development, with Vegfa signaling being found mainly upstream of Notch during specification of angioblasts into arteries. Vegfa activates intracellular signaling, including Notch, to modulate artery formation via different downstream kinases: phosphatidylinositol 3-kinase (PI3K) and extracellular signal-regulated kinase (ERK), a member of the mitogen-activated protein kinase (MAPK) family.

In mouse arteries, Vegfa-stimulation of the MAPK/ERK pathway induces the expression of arterial-specific genes, including delta-like ligand-4 (*Dll4*), the gene encoding its receptor, Notch4, and its target gene, ephrin B2 (*Efnb2*). In contrast, stimulation of the PI3K pathway has been reported to inhibit the ERK-triggered arterial genetic program in favor of venous specification (Ackah et al., 2005; Deng et al., 2013; Liu et al., 2008; Phung et al., 2006; Zhu et al., 2013). Similarly, Notch functions downstream of Vegfa signaling during zebrafish artery development (Lawson et al., 2001), although MAPK/ERK signaling is not required for this process (Shin et al., 2016). Thus, the combination of protein kinase signaling events that mediate Vegfa activation of Notch signaling in zebrafish remains incompletely understood.

The Ser and Thr kinase Akt is a key kinase in the PI3K signaling pathway and regulates important processes in tissue formation, such as growth and survival (Franke et al., 1997). Akt activation leads to the phosphorylation of multiple substrates, enabling Akt to exert diverse downstream effects upon activation by a single upstream growth factor signaling event (Manning and Cantley, 2007). Vertebrates have three Akt protein isoforms encoded by the *Akt1*, *Akt2* and *Akt3* genes, which are highly conserved across species. Chemical inhibitors that modulate PI3K signaling and single or double tissue-specific Akt gene knockouts have allowed examination of Akt function during model organism development (Scheid and Woodgett, 2001). However, mouse embryos carrying double mutations of Akt (*Akt1* and *Akt3* mutants) genes are embryonic lethal (Peng et al., 2003). Thus, it has been difficult to directly test the effect of Akt signaling loss on tissue embryogenesis. Despite the lack of genetic models, Akt signaling has been implicated in the development of artery and vein formation in mice by exerting an inhibitory influence on ERK signaling (Ren et al., 2010) and promoting venous formation via direct phosphorylation of the COUP transcription factor 2 (Nr2f2), a key driver of venous specification (Chu et al., 2016). However, mice globally lacking Akt1, the main isoform expressed in endothelial cells, are viable, smaller in size, and show reduced angiogenesis in the placenta, retina and limbs (Ackah et al., 2005; Ha et al., 2019; Schleicher et al., 2009; Yang et al., 2003). However, conditional loss of Akt1 in the endothelium leads to reduced postnatal arteriogenesis and sprouting of retinal (Lee et al., 2014) and coronary vessels (Kerr et al., 2016). Thus, mouse genetic studies have not established a clear role for Akt signaling during embryonic artery or vein development.

To assess the role of Akt in early embryonic vascular development, we generated a novel loss-of-function model in zebrafish. Using CRISPR/Cas9-mediated deletion, we created loss-of-function mutations in all four zebrafish Akt genes (*akt1*, *-2*, *-3a* and -*3b*), resulting in embryos lacking Akt activity (*akt*^Δ/Δ^). Live-cell imaging coupled with single-cell RNA-sequencing analysis reveal that Akt signaling is not essential for early embryogenesis, but is required for tissue-specific functions, such as the specification of endothelial cell progenitors during the initial formation/remodeling of the main embryonic artery. We found that Akt signaling mediates arterial specification via activation of Notch signaling and functions independently of ERK pathway in this context. Through our analysis, we implicate Akt as a protein kinase that mediates artery specification in the early embryo.

## RESULTS

### Akt signaling is required for tissue-specific rather than general embryonic development

To investigate the role of Akt signaling in development, we generated a Akt loss-of-function model in zebrafish embryos. In zebrafish, as in mammals, Akt proteins contain an N-terminal PH (pleckstrin homology) domain followed by a catalytic (kinase) domain and a regulatory C-terminal tail (C-tail) (Yang et al., 2002). Upon stimulation with growth factors, Akt is recruited to the plasma membrane via its PH domain, where PI3K-dependent phosphorylation of key amino acids (Thr308/Ser473) in the kinase or C-tail domain triggers its catalytic activation (Chu et al., 2020).

**Fig. 1.**
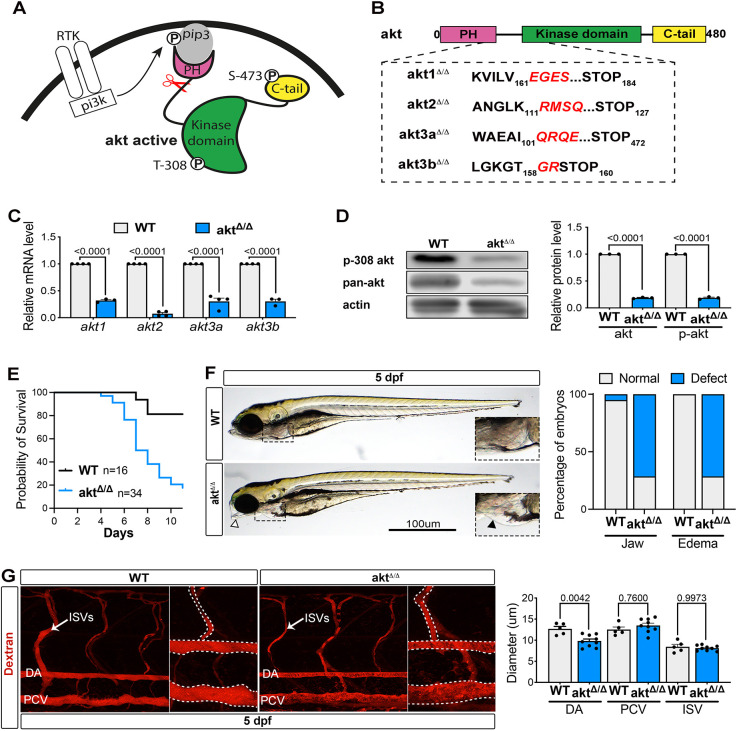
**Zebrafish model of Akt loss of function, *akt*^Δ/Δ^.** (A) Schematic showing Akt activation downstream of receptor tyrosine kinase activation. Scissors represent the site where CRISPR/Cas9 will create mutations. (B) CRISPR/Cas9 strategy. Akt protein structure representation shows protein sequence alignment of wild-type (Akt) and mutant (*akt*^Δ/Δ^) zebrafish lines. Mutated sequences are shown in red. (C) qRT-PCR showing mRNA levels in WT and *akt*^Δ/Δ^ embryos at 4 dpf. Expression levels were normalized to the WT (*n*=3 independent pools of 5 embryos; two-way ANOVA with Šídák's multiple comparisons test). (D) Western blot analysis of WT and *akt*^Δ/Δ^ embryos at 4 dpf (*n*=3 independent pools of 20 embryos; ordinary one-way ANOVA with Tukey's multiple comparison). (E) Survival curve of WT and *akt*^Δ/Δ^ mutants. (F) Left: Brightfield images of zebrafish WT and *akt*^Δ/Δ^ embryos at 5 dpf. Arrowhead shows jaw defect and the boxed area is shown at higher magnification in insets to show edema defects in more detail. Right: Graph shows the percentage of embryos with jaw or edema defects in WT and *akt*^*Δ/Δ*^ *embryos* [*n*=20 (WT), 9 (*akt*^Δ/Δ^) embryos]. (G) Left: Images of live 5 dpf WT or *akt*^Δ/Δ^ embryos injected with tetramethylrhodamine/dextran (2,000,000 molecular weight). The dashed lines indicate the perimeter of the vessels. Graph represents the DA, PCV and ISV diameter quantification [*n*=5 (WT), 9 (*akt*^Δ/Δ^) embryos; Mann–Whitney test]. All quantifications are represented as mean±s.e.m. P, phosphorylation; PH, pleckstrin homology domain; pi3k, phosphatidylinositol 3-kinase; pip3, phosphatidylinositol (3,4,5)-trisphosphate; RTK, receptor tyrosine kinase.

Based on this information, we targeted Akt using CRISPR/Cas9 (Moreno-Mateos et al., 2015) to introduce a premature stop codon within the PH-kinase linker sequence, which is essential for protein kinase activation (Fig. 1A,B). Thus, we multiplexed gRNAs (Narayanan et al., 2016) to simultaneously generate insertions or deletions within the PH-kinase linker (Akt2 and Akt3a) or within the beginning of the kinase domain (Akt1, Akt3b). We then selected germ cell mutations inducing premature stop codon in all four Akt proteins (Fig. 1B).

Although mice lacking all three Akt orthologs cannot be generated, we were able to obtain viable adult zebrafish carrying *akt1*, *-3a* and -3*b* as homozygous and *akt2* as heterozygous mutations. These lines allowed us to generate embryos with homozygous mutations in all Akt isoforms (named *akt*^Δ/Δ^). Compared with wild type (WT), *akt*^Δ/Δ^ embryos had an ∼80% reduction in the mRNA levels of each Akt gene (Hentze and Kulozik, 1999) (Fig. 1C). Furthermore, using antibodies recognizing both endogenous levels of total Akt proteins as well as phosphorylated proteins at Thr308 (Vincent et al., 2011), we found that *akt*^Δ/Δ^ embryos offer a model in which Akt signaling activity is significantly diminished (Fig. 1D).

Notably, we found that *akt*^Δ/Δ^ embryos were indistinguishable from WT during the first 48 h post-fertilization (hpf), when organogenesis is accomplished in zebrafish (Stainier, 2001) (Fig. S1A,B). For example, *akt*^Δ/Δ^ embryos showed normal body length and head size, suggesting that Akt is dispensable for overall embryogenesis (Fig. S1A,B).

Next, we followed the development and survival of zebrafish larvae and found that nearly 90% of *akt*^Δ/Δ^ larvae die by day 11, with a progressive increase in mortality after day 5 (Fig. 1E). In 70% of *akt*^Δ/Δ^ larvae at day 5, we noted a mild craniofacial lower jaw defect and pericardial edema (Fig. 1F). Pericardial edema is commonly found in embryos when the developing vascular system is compromised (Stainier et al., 1996). Thus, we visualized the cardiocirculatory system in *akt*^Δ/Δ^ and WT animals at 5 days post-fertilization (dpf) in more detail using microangiography with high molecular weight rhodamine fluorescent dextran (Weinstein et al., 1995). We first identified no differences in overall structure of the trunk vasculature (Fig. 1G). However, we found that *akt*^Δ/Δ^ mutants had a significantly reduced DA lumen size compared with WT embryos, whereas the PCV and intersegmental vessel (ISV) lumens were unaltered (Fig. 1G). These data suggest that Akt signaling is dispensable for embryogenesis, but required for tissue-specific development such as the vascular system.

### Loss of Akt signaling affects the early embryonic formation of the dorsal aorta

We found that lack of Akt signaling has tissue development defects, affecting, for example, the proper formation of the trunk vasculature, particularly the DA. To unveil the organotypic impact of Akt activity in the embryo, we concentrated, as a proof of concept, on vascular defects in *akt*^Δ/Δ^. Notably, we found that *akt1*, *-2* and *-3b* isoforms were expressed in endothelial cells isolated from zebrafish embryos at 24 hpf when the trunk vascular system (DA, PCV and ISVs) is forming (Gore et al., 2012) (Fig. S2A). *akt1* showed the highest expression of these isoforms, congruent with previous observations in mammalian models (Lee et al., 2014).

**Fig. 2.**
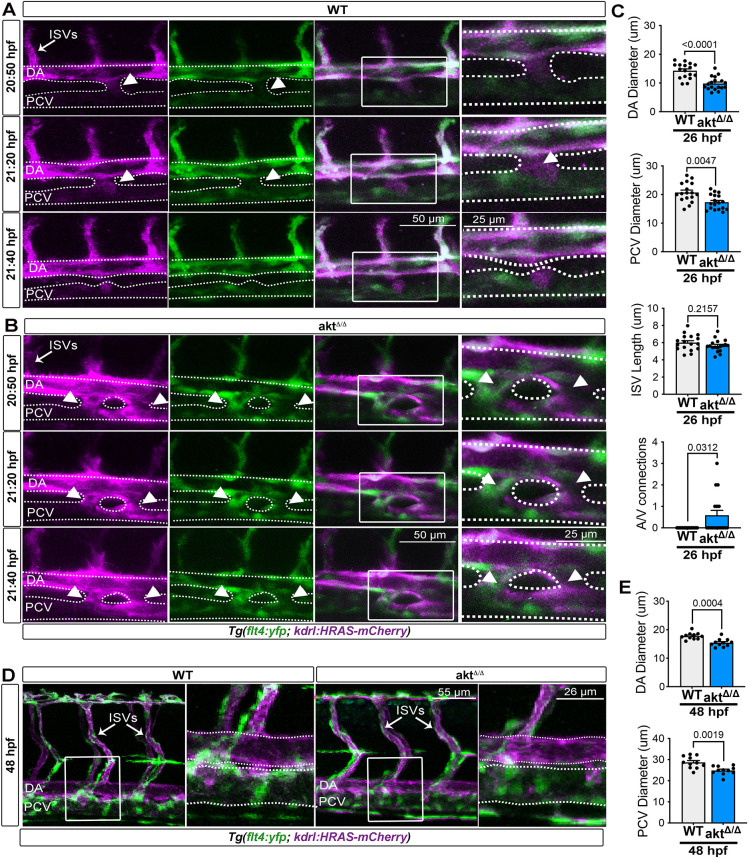
***akt*^Δ/Δ^ embryos exhibit early vascular defects.** (A,B) Images from live time-lapse movies of WT (A) and *akt*^Δ/Δ^ mutants (B) in *Tg (flt4:YFP; kdrl:HRAS-mCherry)^hu4881;s896^* embryos (20-22 hpf). Time stamps are on the left of each panel. White arrowheads indicate connection between the artery and vein. See also Movies 1 and 2. (C) Quantification of DA and PCV diameters and ISV length as well as quantification of artery and vein connections [*n*=17 (WT) and 18 (*akt*^Δ/Δ^) embryos; Mann–Whitney test and Wilcoxon test for A/V connection]. (D) Lateral view (25×) live images of WT and *akt*^Δ/Δ^ embryos at 48 hpf (trunk region) in *Tg (flt4:YFP; kdrl:HRAS-mCherry)^hu4881;s896^*. (E) Quantification of DA and PCV diameters in WT and *akt*^Δ/Δ^ mutants [*n*=17 (WT) and 18 (*akt*^Δ/Δ^) embryos; Mann–Whitney test]. All quantifications are represented as mean±s.e.m. In A.B,D, images on the right are magnified views of the boxed regions to the left, and dotted lines outline vessel perimeters. A/V, artery and vein; DA, dorsal aorta; ISVs, intersegmental vessels; PCV, posterior cardinal vein.

Next, we generated a transgenic reporter line, *flt4:YFP^hu4881^; kdrl:HRAS-mCherry^s896^* (Hogan et al., 2009), in an *akt*^Δ/Δ^ background to allow the analysis of artery (YFP^+^/mCherry^+^) and vein (YFP^+^) formation (Fig. 2A). To document the formation of the vascular system in an *akt*^Δ/Δ^ background during the first 24 hpf, we performed live time-lapse imaging starting from 20 hpf focusing on the trunk region where the main embryonic artery and vein are forming at this stage. In WT embryos, the artery progenitors sprouted ventrally from the DA primordium to form venous cells and segregate into the PCV by 22 hpf (Fig. 2A) (Herbert et al., 2009). Interestingly, in *akt*^Δ/Δ^ mutants, artery progenitors sprouted to form the PCV but failed to separate by 22 hpf (Fig. 2B).

Consistent with the disrupted segregation of artery and vein cells, *akt*^Δ/Δ^ mutants showed a decrease of DA and PCV lumen formation compared with WT at 24 hpf (Fig. 2C). Notably, *akt*^Δ/Δ^ ISVs originating from the dorsal sprouting of the DA endothelial cells grew normally at the same stage (Fig. 2C). Thus, the *akt*^Δ/Δ^ artery and vein development defect was not a general consequence of a delay in vascular development. Furthermore, the reduction of DA and PCV lumen formation was not linked to changes in endothelial cells numbers or obvious endothelial (or non-endothelial) cellular death, because *akt*^Δ/Δ^ and WT strains showed similar numbers of apoptotic cells, as revealed by the TUNEL assay (Fig. S2B).

Next, we examined the DA and PCV around 48 hpf when trunk circulation is fully functional. We found that *akt*^Δ/Δ^ artery and vein cells segregated normally by this time. However, the diameter of the DA, PCV and ISVs were slightly, but significantly, diminished (Fig. 2D,E; Fig. S2C). Congruently, *akt*^Δ/Δ^ mutants at 48 hpf were able to maintain normal circulatory blood flow as the expression of the hemodynamics-sensing genes *klf2*, *pdgfb* and *cxcr4* (Stratman et al., 2020) were similar to WT at this stage (Fig. S2D), suggesting that the capacity of *akt*^Δ/Δ^ endothelial cells to respond to blood flow hemodynamics might resolve early arteriovenous malformations (Corti et al., 2011). Nevertheless, by 5 dpf, only the DA diameter was still affected, suggesting an ongoing effect of Akt loss on this vessel (Fig. 1G).

Overall, these results suggest that Akt signaling contributes to the proper establishment of the embryonic artery and vein layout in the zebrafish trunk, a key step for the formation of the embryonic circulatory system.

### Akt signaling is required for the specification of artery cells independent of ERK phosphorylation

To properly form the main embryonic artery and vein in the zebrafish trunk at 24 hpf, angioblasts migrate from the lateral plate mesoderm (LPM) between the 10 and 18 somite stage (ss) toward the embryonic midline where the expression of *vegfaa* (ortholog of mammalian Vegfa) first instructs arterial specification (Lawson et al., 2002; Zhong et al., 2001). Thus, to identify the cause of early embryo arteriovenous anomalies in *akt*^Δ/Δ^ mutants, we analyzed both the migration of angioblasts and somite *vegfaa* expression.

**Fig. 3.**
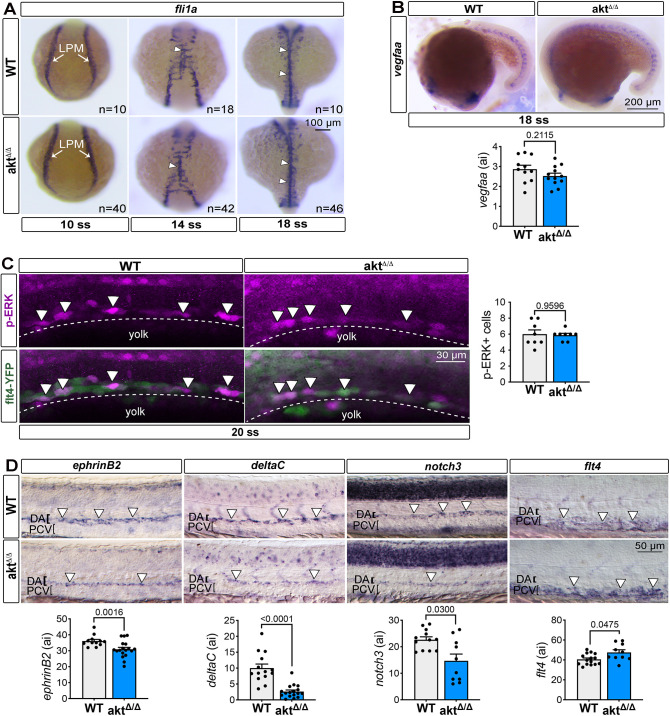
**Loss of Akt impedes artery specification, but not angioblast homing or ERK signaling.** (A) *In situ* hybridization of the marker *fli1a* labels the position of angioblasts at 10 ss, 14 ss and 18 ss in WT and *akt*^Δ/Δ^ embryos. White arrows point to the lateral plate mesoderm (LPM) at 10 ss and white arrowheads indicate the midline formation at 14 ss and 18 ss. (B) Top: *In situ* hybridization of *vegfaa* at 18 ss in WT and *akt*^Δ/Δ^ embryos. Bottom: Graph showing *vefgaa* quantification. All values are divided by 10,000. *n*=11 (WT) and 11 (*akt*^Δ/Δ^) embryos; Mann–Whitney test. (C) Left: Lateral view of immunofluorescence images showing phosphorylated ERK in the DA of WT and *akt*^Δ/Δ^ embryos in *Tg(flt4:YFP)^hu4881^* embryos at 20 ss [*n*=8 (WT) and 8 (*akt*^Δ/Δ^) embryos; Mann–Whitney test]. Dotted lines represent the DA primordium and arrowheads indicate p-ERK^+^ cells. Right: Graph showing the number of p-ERK^+^ cells. (D) Top: *In situ* hybridization of the arterial markers *ephrinB2* [*n*=12 (WT) and 18 (*akt*^Δ/Δ^) embryos], *deltaC* [*n*=14 (WT) and 17 (*akt*^Δ/Δ^) embryos] and *notch3* [*n*=12 (WT) and 10 (*akt*^Δ/Δ^) embryos] and the venous marker *flt4* [*n*=15 (WT) and 10 (*akt*^Δ/Δ^) embryos] at 24 hpf. Arrowheads indicate the region expressing the gene of interest. Bottom: Graph representing all gene expression quantification. All values are divided by 10,000; Mann–Whitney test. All quantifications are represented as mean±s.e.m. ai, arbitrary unit; DA, dorsal aorta; LPM, lateral plate mesoderm; PCV, posterior cardinal vein.

We first investigated whether endothelial progenitors were positive for the angioblast mesenchymal gene *fli1a* (*fli1*) in *akt*^Δ/Δ^ embryos at 10, 14 and 18 ss (Lawson and Weinstein, 2002b). The pattern of *fli1a* cells, as well as the overall intensity of expression, were undistinguishable between WT and *akt*^Δ/Δ^ LPM at all analyzed stages (Fig. 3A). Moreover, given that Akt has been reported to transduce Vegfa signaling (Gerber et al., 1998), we analyzed the expression level of *vegfaa*. Interestingly, the levels of *vegfaa* expression at 18 ss were not altered in *akt*^Δ/Δ^ versus WT embryos (Fig. 3B). Thus, the altered arteriovenous separation in *akt*^Δ/Δ^ embryos was not a consequence of impaired *fli1a*^+^ cell migration or reduced *vegfaa* expression.

Previous studies showed that the PI3K/Akt pathway can enhance venous specification during zebrafish vascular development through negative regulation of the MAPK activity of ERK, downstream of Vegfa (Hong et al., 2006; Ren et al., 2010). To test whether Akt signaling was impairing trunk artery development via modulation of ERK, we stained *akt*^Δ/Δ^ and WT embryos with an antibody recognizing the total p44/42 Map kinase (ERK/ERK) protein (p-ERK) at 20 ss. In accordance with the previous literature (Shin et al., 2016), p-ERK localized to the angioblasts of the DA primordium (Fig. 3C). Interestingly, the number of p-ERK^+^ cells was comparable in WT and *akt*^Δ/Δ^ 20 ss embryos (Fig. 3C). Furthermore, the overall levels of ERK and p-ERK in the embryos, as quantified by western blotting, remained unchanged between WT and *akt*^Δ/Δ^ embryos (Fig. S3A), suggesting that Akt signaling is necessary for the proper formation of arteriovenous vessels, regardless of ERK phosphorylation.

Next, we examined whether improper arteriovenous remodeling in the *akt*^Δ/Δ^ trunk is related to a lack of artery and/or vein cell specification. In fact, the lack of arteriovenous segregation by 21 hpf in *akt*^Δ/Δ^ is reminiscent of the arterial specification defect found in models with reduced Notch-dependent arterial genes (Herbert et al., 2009; Lawson et al., 2002; Quillien et al., 2014). To verify this hypothesis, we tested the expression of the *efnb2a*, *dlc* and *notch3* genes in the DA at 24 hpf. Relative to WT, *akt*^Δ/Δ^ embryos showed a decreased expression of all these arterial markers in the DA at 24 hpf (Fig. 3D). In contrast, the expression of a PCV venous marker, *ftl4*, or other markers for the maturation of spinal cord neurons [*ngn1* (*neurog1*) and *elavl3*; Kim et al., 1996; Korzh et al., 1998] and somites [*myoD* (*myod1*); Weinberg et al., 1996], showed no obvious changes in *akt*^Δ/Δ^ versus WT embryos (Fig. 3D; Fig. S3B).

Thus, arteriovenous defects in *akt*^Δ/Δ^ mutants are associated with the improper specification of artery cells.

### Loss of Akt signaling affects the expression of genes involved in artery, but not vein, specification

The data described above show that *akt*^Δ/Δ^ embryos have decreased expression of artery genes in the DA at 24 hpf. To test this phenotype in more detail at the molecular level, we used fluorescence-activated cell sorting to isolate whole-embryo endothelial cells (*kdrl*:mCherry^+^) from WT and *akt*^Δ/Δ^ embryos at 24 hpf and performed single-cell RNA sequencing (scRNA-seq). We isolated and sequenced 25,565 cells from WT and *akt*^Δ/Δ^ embryos. We first grouped cells into 21 clusters using an unbiased graph-based clustering method and represented the data as uniform manifold approximation and projection (UMAP) graphs (Fig. S4A) (Becht et al., 2019). There were no major differences in cluster distribution or number between WT and *akt*^Δ/Δ^ cells (Fig. S4A), suggesting no major lack or gain of different cell types during vascular development.

**Fig. 4.**
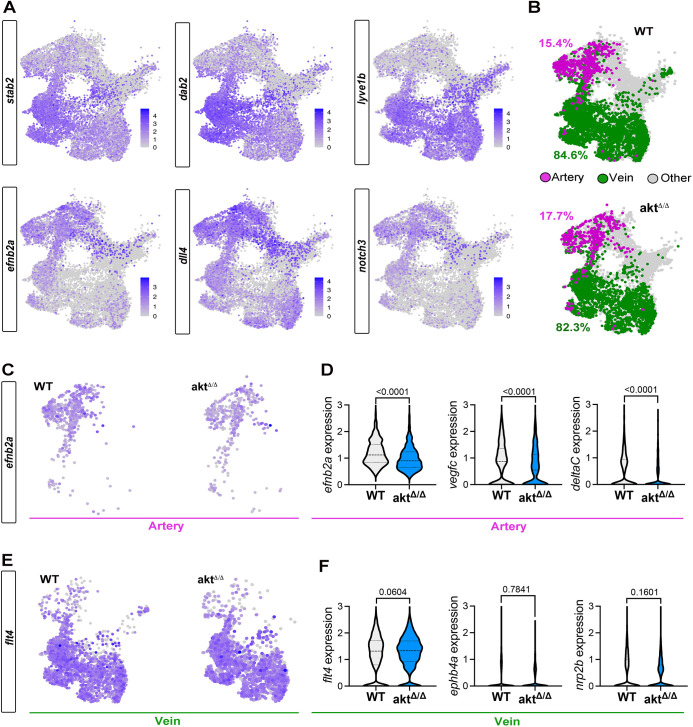
**Single-cell RNA sequencing shows defects in artery specification of *akt*^Δ/Δ^ endothelial cells.** (A) UMAP of venous (*stab2*, *dab2*, *lyve1b*) and arterial (*dll4*, *efnb2a*, *notch3*) markers expressed in all endothelial cell subsets. (B) UMAP of artery and venous classification using our marker strategy identification. The percentages correspond to the proportion of arterial (magenta) or venous (green) cell populations out of all endothelial cell subsets. (C) UMAP of *efnb2a* expression in WT and *akt*^Δ/Δ^ arterial subsets. (D) Violin plot of *efnb2a*, *vegfc* and *deltaC* expression within the arterial subset (Mann–Whitney test). (E) UMAP of *flt4* expression in WT and *akt*^Δ/Δ^ venous subsets. (F) Violin plot of *flt4*, *ephb4a* and *nrp2b* expression within the venous subset (Mann–Whitney test). All quantifications are represented as mean±s.e.m.

To elucidate the identity of each cell in the identified groups, we applied a marker-based approach to define 21 clusters (Fig. S4B). Besides endothelial cells, we also identified hematopoietic stem and progenitor cells (Cluster 15) via the expression of *gata2b* and *myb* (Ghersi et al., 2023), erythroid cells (Cluster 2) via the expression of *gata1a* and *alas2* (Ghersi et al., 2019; Kasper et al., 2020), and possible contaminants such as notochord cells (Cluster 14) expressing *col5a3a* and *cnmd* (Wagner et al., 2018). From the initial 25,565 cells, we then subclustered cells with higher expression levels of pan-endothelial markers such as the endogenous *kdrl* and *cdh5* genes (Fig. S4B) (Larson et al., 2004; Liao et al., 1997). These endothelial cells corresponded to a total of 16,933 cells (Fig. S4B). We next selected cells co-expressing three venous-specific genes, *stab2*, *dab2* and *lyve1b* (named vein), or three artery-specific genes, *dll4*, *efnb2a* and *notch3* (named artery) (Fig. 4A,B) (Gurung et al., 2022). We found that in both WT and *akt*^Δ/Δ^ cells, 82-84% of endothelial cells were veins and 15-17% were arteries (Fig. 4B). This observation aligns with the embryonic stage, when widespread arterialization from venous cells is not yet fully realized in the cranial region (Fujita et al., 2011).

Because the percentage of endothelial cells expressing vein and artery markers was similar across genotypes (Fig. 4B), we searched for differences in the overall level of gene expression. Consistent with the differences detected by whole mount *in situ* hybridization, we observed a significant decrease in the expression level of the arterial markers *efnb2a*, *vegfc* and *dlc* in the *akt*^Δ/Δ^ artery cells compared with WT (Fig. 4C,D). The expression of genes associated with venous specification, such as *flt4*, *ephb4a* and *nrp2b*, were not significantly changed (Fig. 4E,F). Moreover, we observed that the expression levels of *flt4*, *ephb4a* and *nrp2b* remained unaltered in the *akt*^Δ/Δ^ artery cells, and the expression levels of *efnb2a*, *vegfc* and *dlc* in the *akt*^Δ/Δ^ vein cells consistently mirrored those of the WT (Fig. S4C). These findings support the idea that artery and vein cells did not undergo a fate switch, likely explaining why arteriovenous malformations are resolved by the onset of circulation. Altogether, we show that, during formation of the embryonic vessels, artery cells lacking Akt signaling components are not properly specified whereas vein cells form normally.

### Akt signaling controls artery specification upstream of the Notch pathway

Based on our data, artery cells are not appropriately specified in the absence of Akt signaling, causing aberrant artery-vein segregation during development of the DA primordium. This suggests that Akt might have a direct role in the regulation of arterial gene expression independently of other kinases. Consistently, previous studies have shown that Notch acts downstream of Akt signaling in multiple processes, including cancer, neuronal development, or postnatal angiogenesis (Bedogni et al., 2008; Kerr et al., 2016; Konantz et al., 2016; Zhang et al., 2011a). Given that Notch is the master regulator of artery cell specification at this stage of DA development (Quillien et al., 2014), we hypothesized that Akt could sustain the artery gene program via the modulation of Notch activity.

**Fig. 5.**
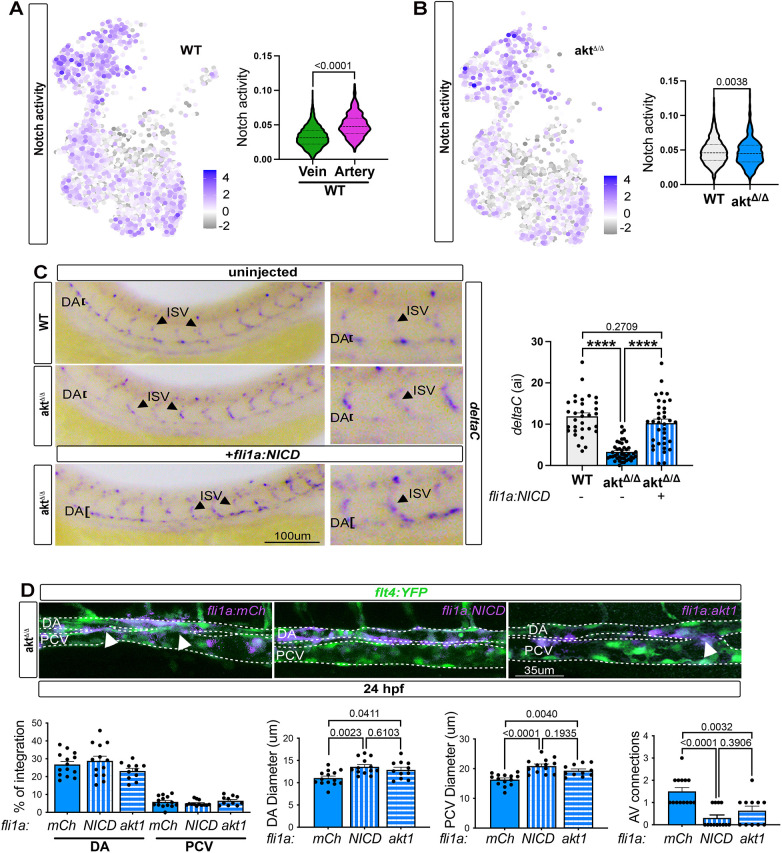
**Loss of Akt alters artery specification via Notch signaling.** (A) UMAP of Notch activity in WT and violin plot of Notch signaling activity in venous and arterial cells, obtained with AUCell (Mann–Whitney test). (B) UMAP of Notch activity in *akt*^Δ/Δ^ venous and arterial cells, and violin plot of Notch activity in WT and *akt*^Δ/Δ^ arterial cells (Mann–Whitney test). (C) *In situ* hybridization against *deltaC* in WT and *akt*^Δ/Δ^ injected or not with endothelial cell-specific NICD (+*fli1a*:*NICD*) at 24 hpf [*n*=32 (WT), 43 (*akt*^Δ/Δ^) and 35 (*akt*^Δ/Δ^ +*fli1a*:*NICD*) embryos; ordinary one-way ANOVA]. (D) Top: Live imaging of *akt*^Δ/Δ^ embryos at 24 hpf injected or not with *fli1a:mCherry*, *fli1a*:*akt1* or *fli1a:NICDi*. Arrowheads indicate connection between artery and vein. Dashed lines delineate the DA and PCV. Bottom: Quantification of the percentage of integration of each construct in the DA or PCV, DA and PCV diameters, and A/V connections [*n*=14 (*akt*^Δ/Δ^ +*fli1a*:*mCh*), 13 (*akt*^Δ/Δ^ +*fli1a*:*NICD*) and 11 (*akt*^Δ/Δ^ +*fli1a*:*akt1*) embryos; ordinary one-way ANOVA]. All quantifications are represented as mean±s.e.m. DA, dorsal aorta; PCV, posterior cardinal vein.

To test this hypothesis, we first interrogated how Notch activity is regulated in the *akt*^Δ/Δ^ artery cells identified in our single-cell sequencing analysis. We used a previously developed algorithm, named AUCell, which scores the activity of signaling networks in each cell by assessing the entire Notch signaling gene set, and enables comparison across genotypes, making it possible to identify cells with distinct Notch signaling activity (Aibar et al., 2017). We first identified Notch-active WT cells by sampling and scoring the expression of the Notch-dependent genes reported under the zebrafish Kyoto Encyclopedia of Genes and Genomes (KEGG) pathway. We identified that the Notch-active cells were significantly enriched in our artery versus vein cells (Fig. 5A). Next, we evaluated the level of Notch-active cells in *akt*^Δ/Δ^ compared with WT artery cells. Consistent with our prediction, artery cells lacking Akt signaling exhibited significantly less Notch activity (Fig. 5B). We also used the same approach to verify that Mapk activity was not diminished in the same *akt*^Δ/Δ^ artery cells (Fig. S5A,B).

To verify at the functional level whether endothelial Notch activity could restore arterialization and proper artery and vein lumen formation in *akt*^Δ/Δ^, we independently activated Notch under the *fli1a* endothelial promoter. We cloned the Notch1 intracellular domain (NICD) (Quillien et al., 2014) and utilized the Tol2-transposon system to introduce *fli1a*:NICD into *akt*^Δ/Δ^ mutants in ∼18-45% of DA ECs (Fig. 5D) and confirmed expression of the artery genes *dlc* and *ephrinB2*. Remarkably, we found that both *dlc* and *ephrinB2* levels were reduced in the *akt*^Δ/Δ^ artery cells (Figs 3D, 4D, 5C; Fig. S5C), but the endothelial activation of NICD was adequate to restore their expression in the mutant DA to a level similar to that of WT embryos at 24 hpf (Fig. 5C; Fig. S5C). No difference was noted in the expression of *dlc* in other vessels, such as the ISVs (Fig. 5C), supporting the notion that Akt-Notch regulation occurs in the main embryonic artery. Interestingly, the expression of NICD in artery cells was sufficient to ameliorate the effect on PCV diameter, suggesting that the early vascular venous diameter defect is a potential consequence of improper artery specification (Fig. 5D). This was further corroborated by the expression of a constitutively active form of Akt1, one of the main endothelial-specific isoforms, in the DA. This intervention was adequate to enhance DA and PCV diameter and arteriovenous connections compared with the control fli1a:mCh alone (Fig. 5D).

**Fig. 6.**
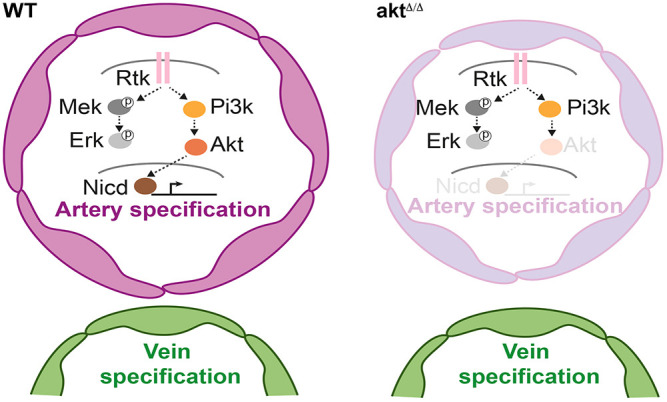
**Model of artery specification controlled by Rtk/Akt/Notch signaling.** In this study, we created the first zebrafish model with loss-of-function mutations in all four Akt genes (*akt*^Δ/Δ^). We found that Akt signaling is required for specifying endothelial cell progenitors into arteries independently of other kinases. Notably, restoring Notch activity in the dorsal aorta compensates for Akt loss, suggesting that Akt acts upstream of Notch in artery specification during embryonic development. Pi3k, phosphatidylinositol 3-kinase; Rtk, receptor tyrosine kinase.

Overall, these data suggest that Akt signaling is required during the development of the DA upstream of Notch signaling to regulate early arterial specification (Fig. 6).

## DISCUSSION

Despite the central role of Akt signaling in vascular homeostasis (Chu et al., 2016; Ren et al., 2010), its importance during early vascular development has yet to be thoroughly addressed owing to the lack of viable vertebrate genetic models lacking Akt signaling in the embryo. Here, we generate the first vertebrate model, in zebrafish, carrying loss-of-function mutations in all four Akt genes. We first found that embryos lacking pan-Akt signaling undergo embryogenesis normally but manifested tissue-specific developmental defects around 5 dpf. The most prominent anomalies observed were related to vascular artery hypoplasia and lower jaw development, defects that likely compromise the survival of larvae lacking Akt signaling. We examined the phenotype associated with artery development and determined that Akt is required for the proper specification of endothelial progenitor cells into arteries. Single-cell sequencing of endothelial cells from developing artery and vein tissue showed that Akt signaling is required to sustain Notch activity. Notch-dependent artery genes can be restored without Akt signaling in the DA by ligand-independent activation of Notch. Overall, our data suggest that, during the establishment of the embryonic artery, Akt is a kinase upstream of the Notch pathway that sustains artery specification (Fig. 6).

Akt plays a crucial role in the regulation of metabolism, transcription, cell migration, cell cycle progression, and cell survival. The existence of viable knockout mice for each of the three Akt isoforms suggests their functional redundancy. *Akt1*-null mice are growth retarded, *Akt2*-deficient mice develop type 2 diabetes-like syndrome, and *Akt3*-null mice show impaired brain development (Chen et al., 2001; Cho et al., 2001; Tschopp et al., 2005). The double knockout *Akt1*^−/−^ *Akt3*^−/−^ causes embryonic lethality at around embryonic day 11, with severe developmental defects in the cardiovascular and nervous systems (Yang et al., 2005). Double *Akt1*^−/−^ *Akt2*^−/−^ mutants die a few hours after birth and display impaired skin development, skeletal muscle atrophy, and impaired bone development (Peng et al., 2003). Because of the interdependence between the vascular system and other organs and tissues during development, it is difficult to fully dissect which tissue is directly affected by the loss of Akt signaling or the loss of a proper circulatory system in the embryo. In contrast to mice, zebrafish organogenesis and tissue development rely largely on oxygenation from water, rather than from oxygenated red blood cells distributed by the blood vascular circulation. Thus, it is not surprising that our Akt mutants can be analyzed despite having a defect in establishment of the early circulatory vascular loop, i.e. the segregation of artery and vein. Nevertheless, consistent with the mouse model lacking two of the three Akt isoforms (Akt1 and Akt2), we did observe impaired bone development in the craniofacial region in *akt*^Δ/Δ^ embryos (Peng et al., 2003). Furthermore, even if we did not observe major defects in the development of neuronal cells and muscles, we cannot exclude the possibility of specific phenotypes in the function of these tissues, such as neurobehavioral and/or motor control defects. However, our established zebrafish Akt-signaling mutants provide the unprecedented possibility of screening for the physiological roles of Akt kinases throughout the development of other tissues.

Here, we decided to use our Akt signaling loss-of-function embryos to study the physiological role of Akt in the development of the vascular system. Although other studies have attempted to approximate the role of the kinase Akt during vascular development in zebrafish, they mainly induced loss of Akt activity indirectly by inhibiting the upstream kinase function of PI3K (Hong et al., 2006; Lange et al., 2022; Nicoli et al., 2012), which might affect downstream signaling pathways other than Akt phosphorylation and activation. Thus, we reasoned that any discrepancies among zebrafish and/or mouse models analyzing Akt function might be related to the previous lack of a comprehensive genetic model to test Akt function.

Our data particularly focus on the establishment of the main embryonic artery in zebrafish development and we discovered that Akt mutants suffer from improper segregation of artery and vein cells during the formation of the DA. Notably, we did not observe angiogenesis defects associated with the formation of the ISVs, suggesting that other kinases might be required for early angiogenesis downstream of vascular endothelial growth factors. For example, ERK could be downstream of Vegfa-mediated angiogenesis of ISVs, as previously reported (Shin et al., 2016). We found that ERK phosphorylation is unperturbed in zebrafish lacking artery specification, supporting a model in which Akt is the main kinase downstream of Vegfa for artery specification, but not angiogenesis, at this stage of development.

Our data demonstrate that Akt is the kinase upstream of Notch-dependent arterial specification of angioblasts forming the early embryonic artery. Several Notch-dependent arterial genes were downregulated in *akt*^Δ/Δ^ artery cells, and overexpression of NICD restored the expression of the artery fate marker *dlc*. In this context, Akt might regulate Notch activity directly or indirectly. For example, it has been reported that the zinc finger transcription factor Evi1 (Mecom) can induce Notch activity by indirectly sustaining the phosphorylation of Akt during the endothelial-to-hematopoietic transition in the zebrafish DA (Konantz et al., 2016). Furthermore, during postnatal angiogenesis it has been shown that Akt can directly phosphorylate Notch4-ICD (Lee et al., 2014) and regulate Notch4 nuclear localization (Ramakrishnan et al., 2015). Furthermore, Angiopoietin 1 can induce vascular quiescence in endothelial cells by activating Akt signaling and sustaining the level of intracellular Notch indirectly by phosphorylating glycogen synthase kinase 3β (GSK3β), thereby enhancing β-catenin activity (Zhang et al., 2011). Overall, our data and the Akt loss-of-function model system will open the way to exciting new hypotheses on how Akt can directly or indirectly control the level of Notch activity in the main embryonic artery.

Miransertib is a pan-Akt inhibitor used in preclinical studies for PI3K-driven tumors (Yu et al., 2015), and the application of this drug is being evaluated in the treatment of other disease models, such as vascular malformations (Kobialka et al., 2021 preprint). The study presented here shines light on the physiological requirement for Akt signaling in tissue formation during embryonic development. Furthermore, it will also inform on the potential effects of pharmaceutical targeting of Akt signaling during disease states in which embryonic-like programs, including tissue formation, repair, and cancer progression, can be reactivated (Fazilaty and Basler, 2023). Hence, we believe that our newly established zebrafish Akt loss-of-function model, together with our analysis of the vascular system in this mutant, will help to screen for physiological processes linked to Akt signaling and potential undesirable effects of pan-Akt-inhibitor therapies.

## MATERIALS AND METHODS

### Generation of Akt loss-of-function zebrafish

A multiplexed pool of CRISPR sgRNA and Cas9 mRNA were used to generate Akt4 isoform loss-of-function zebrafish. Those fish were generated in the background of *Tg(flt4:YFP; kdrl:mCherry)^hu4881s916^*. gRNA sequences are listed in Table S1. The T7 endonuclease I (NEB) method was used to determine the mutagenesis. Genomic DNA was isolated from a clutch of injected embryos with 100 mM sodium hydroxide and 1 M pH 7.5 Tris-HCl and then amplified regions, including the mutation site, were amplified by PCR. After 3 months, the F0 founder fish was genotyped using fragment analysis (DNA analysis Facility, Yale University, CT, USA) and out-crossed with WT. Genotyping primers are listed in Table S1. After genotyping out-crossed F1 zebrafish, genomic DNA was isolated and amplified with PCR. The PCR products were cloned into TOPO vector with the TOPO TA cloning kit (Invitrogen) for sequencing. The sequencing results demonstrated that the mutant strains were *akt1^ya348^* (−1Δ), *akt2^ya349^* (+10Δ), *akt3a^ya350^* (−21Δ) and *akt3b^ya351^* (−4Δ), which are termed *akt1*^Δ/Δ^, *akt2*^Δ/Δ^, *akt3a*^Δ/Δ^ and *akt3b*^Δ/Δ^ here, with the combination of all four alleles termed *akt*^Δ/Δ^. For Figs 1D, 3B and Figs S3A, S5C, *akt*^Δ/Δ^ embryos were not genotyped, and were generated from the in-cross of *akt1*, *akt3a*, *akt3b* homozygous and *akt2* heterozygous mutants.

### Flow cytometry and qRT-PCR

WT and Akt loss-of function zebrafish embryos were collected at 24 hpf, dechorionated and placed in 2 ml tubes with egg water. Egg water was removed and replaced with 250 µl Dulbecco's phosphate-buffered saline (DPBS). To each tube, 30 µl liberase was added and incubated at 28°C for 45-60 min total. Every 10-15 min, samples were pipetted up and down to disassociate cells. Then, 600 µl 1 mM EDTA was added into each tube and then 200 µl cold fetal bovine serum. Samples were kept on ice and mixed with 1 ml cold suspension media [50 ml suspension media contains 40 µl of 1 M CaCl_2_, 500 µl penicillin 50 units/ml and streptomycin 0.05 mg/ml (Thermo Fisher Scientific), 250 µl fetal bovine serum and 49.21 ml of Leibovitz medium (Sigma-Aldrich)]. The same samples were combined in one 15 ml tube and centrifuged at 1800 rpm (304 ***g***) for 5 min at 4°C. The supernatant was removed and replaced with 2 ml suspension media, then the tube was mixed gently and centrifuged at 1800 rpm (304 ***g***) for 5 min at 4°C. The supernatant was then removed and replaced with 1-2 ml suspension media. The re-suspended pellets were strained into a FACS tube, mixed with 1:2000 of 200 µg/ml DAPI and placed on ice.

Samples were centrifuged at 1800 rpm (304 ***g***) for 5 min at 4°C and mixed with 500 µl TRIzol (Ambion), then100 µl chloroform was added into the samples, followed by 15 s vortexing and 2-3 min incubation at room temperature. After 15 min, samples were centrifuged at 12,000 ***g*** at 4°C, and the aqueous phase was carefully transferred into a new tube and mixed with 250 µl 100% isopropanol. Samples were incubated at −20°C overnight and centrifuged at 12,000 ***g*** for 10 min for 4°C. The pellet was washed with 75% ethanol after removing the supernatant. After the RNA pellet was dry, RNA was re-suspended in water and measured using a NanoDrop One (Thermo Fisher Scientific) machine to determine the concentration and A260/A280 ratio. Finally, 300 ng RNA was mixed with iScript Reaction mix and reverse transcriptase (Bio-Rad) to generate cDNA in a 20 µl reaction mixture for each sample, into which 80 µl water was added; 5 µl out of the resulting 100 µl volume was mixed with SYBR Green master mix (Bio-Rad) for qPCR. The 2^−ΔCT^ and 2^−ΔΔCT^ methods were used to analyze the relative mRNA expression level of genes of interest.

### Western blotting

WT and Akt loss-of function zebrafish embryos (24 h and 4 dpf) were placed in 1.5 ml tubes containing 1× PBS. Embryos were pipetted with a 20 µl tip to break the zebrafish tissues and centrifuged at 1300 rpm (159 ***g***) for 10 min. After removing the supernatant, pellets were re-suspended in lysis buffer (see next paragraph for composition) with 2 µl per embryo. The lysates were left on ice for 15 min, mixed for 15 s and centrifuged at 12,000 rpm (15,523 ***g***) for 10 min at 4°C. The supernatant was transferred to new cold tubes, sonicated for 6 s and protein concentration was measured using a DC Protein Assay kit (Bio-Rad). The samples were mixed with 6× protein buffer and boiled for 5 min. Approximately 20 µg of protein samples were added into each well of a 10% SDS–polyacrylamide gel electrophoresis (SDS-PAGE) gel and run at 80 V for 20 min then 100 V for about 1 h. Then, the SDS-PAGE gel was transferred to 0.45-μm nitrocellulose membranes (Bio-Rad) at 100 V for 2 h. The nitrocellulose membrane was incubated with blocking buffer (Tris-buffered saline with 0.1% Tween 20 with 5% bovine serum albumin) for 1 h, then with primary antibody at 4°C overnight, washed with TBST (Tris-buffered saline with 0.1% Tween 20) for 5 min three times. Blots were visualized using a LI-COR Odyssey imaging system and analyzed using Image Studio software (LI-COR).

Lysis buffer contains 50 mM Tris-HCl, 1% NP-40 (v/v), 0.1% SDS, 0.1% deoxycholic acid, 0.1 mM EDTA, 0.1 mM EGTA and 8 mg NaF, 4 mg sodium pyrophosphate, 20 mg Complete Protease Inhibitors (Roche), 3 mg Pefabloc SC AEBSF (Roche), 0.25 mM sodium orthovanadate and 50 mM β-glycerophosphate in 10 ml lysis buffer. Protein buffer contains 70 ml Tris-HCl, 36 ml glycerol, 10 g SDS, 6 ml 2-methylbutane, 40 mg Bromophenol Blue and water to a final volume of 120 ml. Primary antibodies (1:1000) were: monoclonal rabbit phospho-Akt T308 (Cell Signaling Technology, 2965), monoclonal mouse pan-Akt (Cell Signaling Technology, 2920), monoclonal rabbit p-ERK (Cell Signaling Technology, 4370), monoclonal rabbit ERK (Cell Signaling Technology, 4695) and monoclonal mouse anti-actin (Sigma-Aldrich, A4700). Secondary antibodies (1:10,000) were: goat anti-rabbit Alexa Fluor 680 (Invitrogen, A20984), goat anti-mouse Alexa Fluor 680 (Invitrogen, A21057) and goat anti-mouse DyLight 800 (Rockland, 610-145-002-0.5). Raw images of western blots can be found in Fig. S6.

### *In situ* hybridization and immunofluorescence

For *in situ* hybridization, WT and Akt loss-of-function embryos (10 ss, 14 ss, 18 ss and 24 hpf) were fixed with 4% paraformaldehyde (PFA), pH 7.5, at 4°C overnight. After removing PFA, samples were washed with PBSTw (PBS with 0.02% Tween 20) for 5 min three times and incubated in 100% methanol (MeOH) at −20°C overnight. Samples were washed with 75% MeOH/PBSTw, 50% MeOH/PBSTw, 25% MeOH/PBSTw and PBSTw for 5 min, respectively, and additionally washed with PBSTw three times for 5 min each. Then, samples were digested in proteinase K at 10 µg/ml for 10 min and re-fixed in 4% PFA for 20 min at room temperature, then washed quickly with PBSTw three times. After removing PBSTw, samples were mixed with 300-400 µl HB4 and incubated at 60°C for 1 h. HB4 contains 50% formamide, 25% 20× SSC, 5 mg/ml torula, 50 µg/ml heparin, 0.1% Tween 20 and 5% dextran sulfate. After removing HB4, samples were incubated with 100-150 µl probe in HB4 [*vegfaa* (Lawson et al., 2002); *flt4*, *klf2*, *pdgfrB*, *cxcr4* (Kasper et al., 2017); *notch3*, *ephrinB2*, *deltaC*, *fli1a* (Lawson et al., 2001); *ngn1*, *elavl3* (Kim et al., 1996); *myoD* (Weinberg et al., 1996); 1:300] overnight at 60°C. The next day, samples were washed for 30 min twice with 2× SSCTw/50% formamide at 60°C, once for 15 min with 2× SSCTw and for 30 min twice with 0.2× SSCTw (2× SSCTw contained 2× SSC with 0.1% Tween 20; 0.2× SSCTw contained 0.2× SSC with 0.1% Tween 20). After blocking samples with 300-400 µl 5% sheep serum in PBSTw for 1 h at room temperature with gentle shaking, samples were incubated with 100-150 µl anti-Digoxigenin-AP-Fab fragment (1:10,000) (Roche, 11093274910) in 5% sheep serum with PBSTw at 4°C overnight. At Day 3, samples were washed with PBSTw with variable times starting with 5 min, to 20 min to overnight. After washing for 5 min twice with fresh and filtered staining buffer, samples were incubated in 500 µl staining solution for up to 48 h in dark. To stop the reaction, samples were washed with PBS/20 mM EDTA three times and kept at 4°C. Staining buffer contained 100 mM NaCl, 50 mM MgCl_2_, 100 mM Tris (pH 9.5) and 0.1% Tween 20. Staining solution contained 200 µl NBT/BCIP (Roche) and 9.8 ml staining buffer. Quantification was carried out as described in a previous publication (Dobrzycki et al., 2018).

For immunofluorescence, WT and Akt loss-of-function embryos (24 hpf) were fixed and washed under the same conditions as *in situ* hybridization. After permeabilization with 0.125% trypsin in PBS on ice for 20 min, samples were washed with PBSTw for 5 min three times and blocked at 4°C for 3-4 h. Primary antibodies were: monoclonal rabbit phospho-ERK (Cell Signaling Technology, 4695; 1:100), chicken polyclonal anti-GFP (Abcam, 13970; 1:300) and rabbit polyclonal RFP antibody (antibodies-online, ABIN129578; 1:300) were mixed with samples overnight. Samples were washed six times for 45 min each wash and incubated with secondary antibodies [goat anti-chicken Alexa Fluor 488 and goat anti-rabbit Alexa Fluor 680 (Invitrogen, A-21209 and A-21209; 1:300)] overnight and washed with PBSTw six times for 45 min each wash. Embryos were imaged with a Lecia SP8 microscope.

### TUNEL assay

Thirty to forty WT and Akt loss-of-function zebrafish were placed in 1.5 ml tubes and fixed with 4% PFA at 4°C overnight. Samples were washed with PBS three times, placed in 100% MeOH at −20°C overnight and washed with 75% MeOH/PBSTw, 50% MeOH/PBSTw, 25% MeOH/PBSTw and PBSTw for 5 min, respectively. After treatment with 10 µg/ml proteinase K at room temperature for 1.5 min, samples were washed with PBTw (1× PBS + 0.1% Tween 20) twice and re-fixed in 4% PFA at room temperature for 20 min. Samples were washed with PBST for 5 min five times and incubated with pre-chilled ethanol:acetic acid (2:1) at −20°C for 10 min. After three washes of PBST for 5 min each, samples were incubated at room temperature in 75 µl equilibration buffer for 1 h (ApopTag Red *in situ* Apoptosis Detection kit; Millipore). A small volume of working strength TdT (70% reaction buffer and 30% TdT with 0.3% Triton X-100) was added into the reaction and incubated at 37°C overnight. The positive control was incubated with DNaseI overnight. The negative control was incubated without TdT. The reaction was stopped with working strength stop/wash buffer (1 ml concentrated buffer with 34 ml dH_2_O) for 3-4 h at 37°C. After washing with PBST three times for 5 min each, samples were blocked with 2 mg/ml bovine serum albumin and 5% sheep serum in PBST for 1 h at room temperature and incubated in the dark for 45-60 min at room temperature with working strength rhodamine antibody solution (from the kit). After a 30 min wash with PBST four times, samples were incubated for 15 min with DAPI (1:200) in PBSTw and washed three times with PBST prior to imaging.

### Live fish imaging and size measurement

WT and Akt loss-of-function embryos (24 hpf) were placed in 1% low range agarose gel plate and 0.003% 1-phenyl-2-thiourea, 1% tunicamycin (Millipore-Sigma, T7765) in egg water and imaged by confocal microscopy (Leica SP8 upright confocal microscope). Using the software Neulicuda, we tracked the DA, PCV and ISV manually and then DA, PCV diameters and ISV length were quantified.

### Microangiography and Imaris measurement

Embryos were anesthetized in 0.1% tricaine methane sulfonate, placed on an agarose mold, and injected pericardially with 4 nl tetramethylrhodamine/dextran (2,000,000 molecular weight, Thermo Fisher Scientific) at a concentration of 10 mg/ml. Subsequently, the vasculature of embryos was checked for dextran fluorescence signal under a stereomicroscope (Olympus, MVX10). Embryos with dextran fluorescence were mounted in 1% low-melt agarose within glass-bottom microwell dishes and imaged with a Leica SP8 upright confocal microscope. Confocal fluorescence images were analyzed with Imaris microscopy image analysis software (Bitplane, Oxford Instruments). Vessel diameters were manually traced with the ‘Filament’ module.

### Plasmid expression constructs

The endothelial-specific expression of mCherry and NICD was constructed via a Gateway cloning strategy using pME-mcherry (Addgene #26028) and pentr-NICD (Addgene #46048), p5E-fli1a (Addgene #31160) and pTol2 destination vector.

The endothelial-specific expression of myr-Akt1 was constructed via a Gateway cloning strategy using myr-Akt1 (Chan et al., 2002; Ramaswamy et al., 1999), p5E-fli1a (Addgene #31160) and pTol2 destination vector. Those plasmids were combined in LR multisite Gateway cloning reaction. Embryos were then injected in the one-cell state with 25 pg of the expression construct and Tol2 transposase mRNA, and later selected for mCherry expression.

### scRNA-seq analysis

The single-cell experiment was performed following the 10x Genomics single-cell protocols and with a commercial kit (10x Genomics Chromium Next GEM Single Cell 3′ Library Construction Kit v2.1, PN-120237). Quality control was ensured as previously described (Ghersi et al., 2023; Satija et al., 2015; Stuart et al., 2019). Briefly, the data were filtered such that all the cells that had fewer than 200 unique transcripts detected were eliminated, as were the genes that were seen in fewer than three cells. The data were first clustered in Seurat. Then, we used three markers to define two main clusters as vein and artery.

AUCell (version 1.14.0) was employed for all cells. The area under the curve was used to quantify and test the signature enrichment of the Notch gene set in each cell. The gene sets used were described in the KEGG for both Notch (dre04330) and Mapk (dre04010). All the downstream visualization and analysis were carried out in R language.

### Statistics and reproducibility

The visualizations were generated using ggplot2 (version 3.3.5) and GraphPad Prism (version 9). All sample sizes and statistical tests used are specified in each legend. Prior to the experiments, a power analysis was performed to determine the sample size needed. A Mann–Whitney test was used when comparing two groups to test mean differences (whole-mount *in situ* hybridization, live imaging, immunofluorescence and scRNA-seq). Ordinary one-way ANOVA with Tukey's multiple comparison was used when comparing more than two groups to test mean differences (whole-mount *in situ* hybridization quantification). One-sample *t*-test and Wilcoxon test were used to compare two-paired samples (qRT-PCR). The operator performing quantification was unaware of the sample group for each image. Data distribution was assumed to be normal, but this was not formally tested.

## Supplementary Material



10.1242/develop.202727_sup1 Supplementary information

Table S2. Source data for all experiments part of this study.
