# Supplementary material for: Akt is a mediator of artery specification during zebrafish development
Source: Development. 2024 Sep 2;151(17):dev202727. doi: 10.1242/dev.202727 (PMC11441982; doi:10.1242/dev.202727)
Supplement: Supplementary information [file develop-151-202727-s1.pdf]

Fig.S1

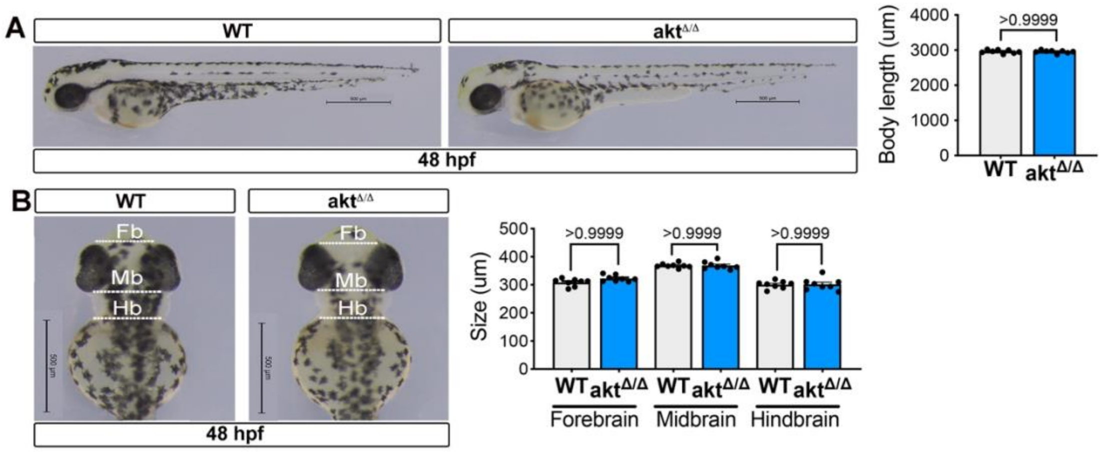

Fig. S1. Gross morphology is not affected in akt$^{\Delta/\Delta}$ zebrafish.

(A) Lateral brightfield images of WT and akt$^{\Delta/\Delta}$ at 48 hpf. Quantification corresponds to head to tail fin measurements. (n= 8 (WT) and 8 (akt$^{\Delta/\Delta}$) embryos, ordinary one-way ANOVA) (B) Dorsal brightfield images of WT and akt$^{\Delta/\Delta}$ heads at 48 hpf. Quantification corresponds to forebrain, midbrain and hindbrain measurements. (n= 8 (WT) and 8 (akt$^{\Delta/\Delta}$) embryos, ordinary one-way ANOVA). All quantifications are represented with mean ± s.e.m. Fb: forebrain, Mb: midbrain, Hb: dpf: hindbrain and hpf: hours post fertilization.

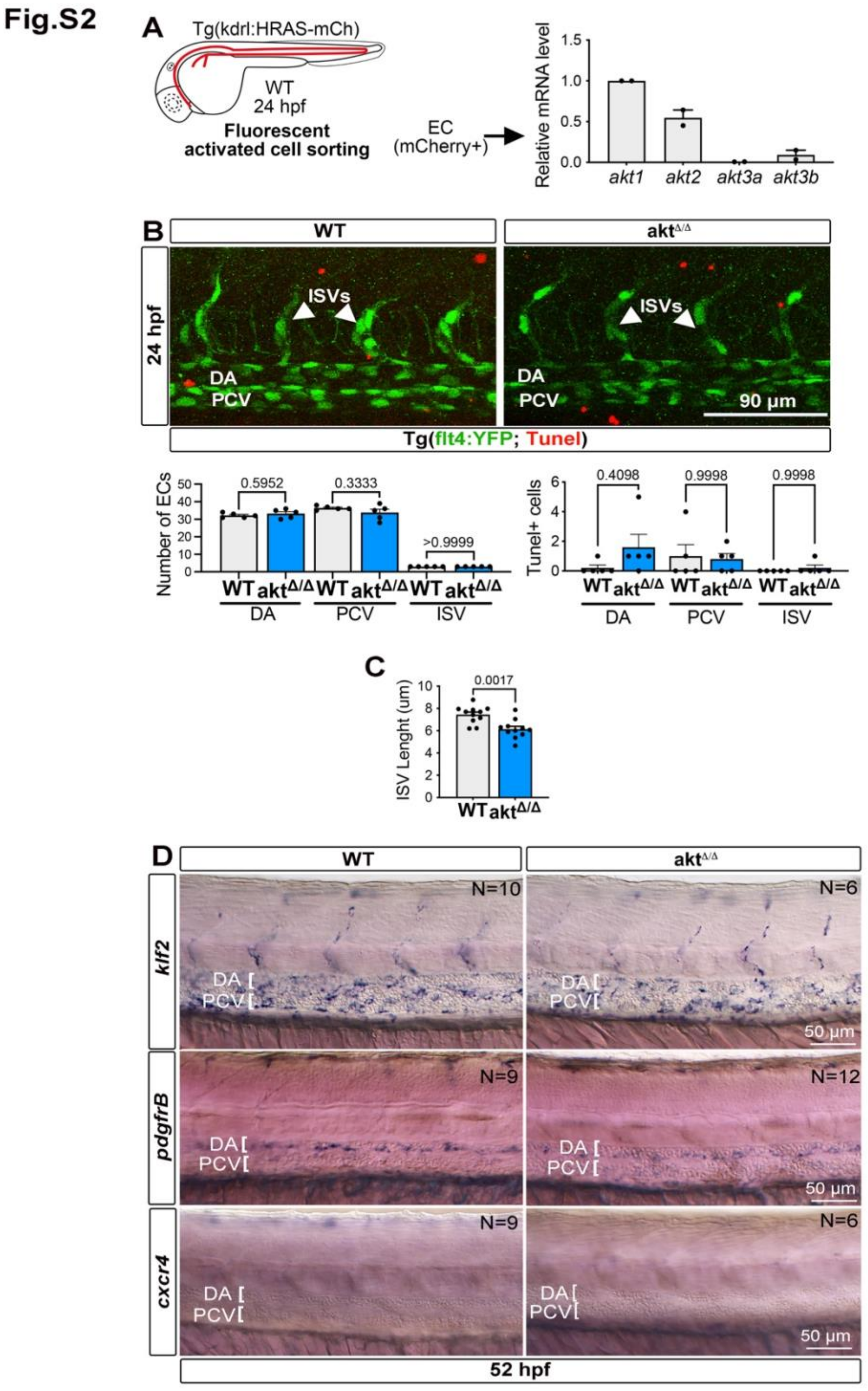

Development • Supplementary information

Fig. S2. Blood flow is not affected in akt$^{\Delta/\Delta}$ zebrafish.

(A) Schematic representation of endothelial cell isolation experiment design. qRT-PCR showing mRNA levels of akt isoforms in WT embryos in endothelial cells (mCherry+) fluorescent activated cell sorted. Expression levels were normalized to akt1 (n= 2 independent experiments constituted of a pool of 5 embryos). (B) Confocal image of WT or akt$^{\Delta/\Delta}$ embryos at 24 hpf trunk with TUNEL assay labeling the apoptotic cells and quantification. (n= 5 (WT) and 5 (akt$^{\Delta/\Delta}$) embryos, ordinary one-way ANOVA). (C) Quantification of intersegmental vessels length from Figure 2C. (D) Whole mount *in-situ* hybridization of *klf2*, *pdgfrB* and *cxcr4* in WT and akt$^{\Delta/\Delta}$ at 52 hpf. All quantifications are represented with mean ± s.e.m. ISVs: intersegmental vessels, DA: Dorsal aorta and PCV: posterior cardinal vein.

Development • Supplementary information

Fig.S3

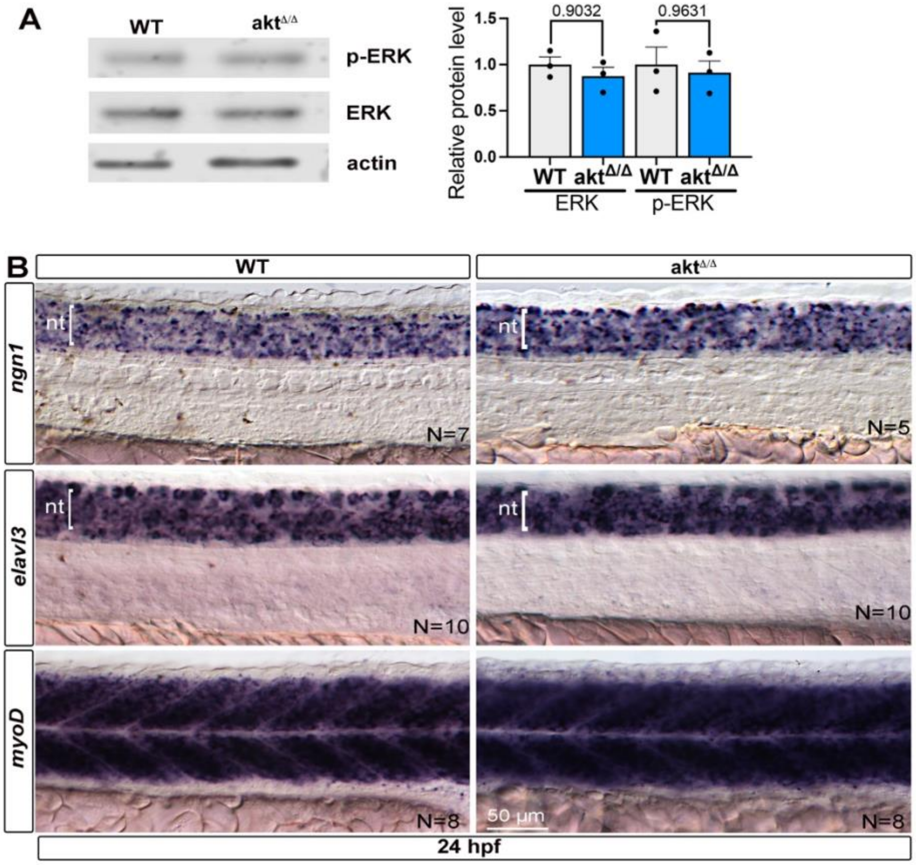

Fig. S3. Notochord and neural tube are not altered in a kt$^{\Delta/\Delta}$.

(A) Western-blot against ERK, p-ERK and actin at 24 hpf WT and akt mutant embryos. (n= 3 independent pools of 50 embryos, ordinary one-way ANOVA with Tukey's multiple comparison). (B) Whole mount *in-situ* hybridization of *ngn1*, *elavl3*, *myoD* expression in WT and akt$^{\Delta/\Delta}$ at 24 hpf.

Development • Supplementary information

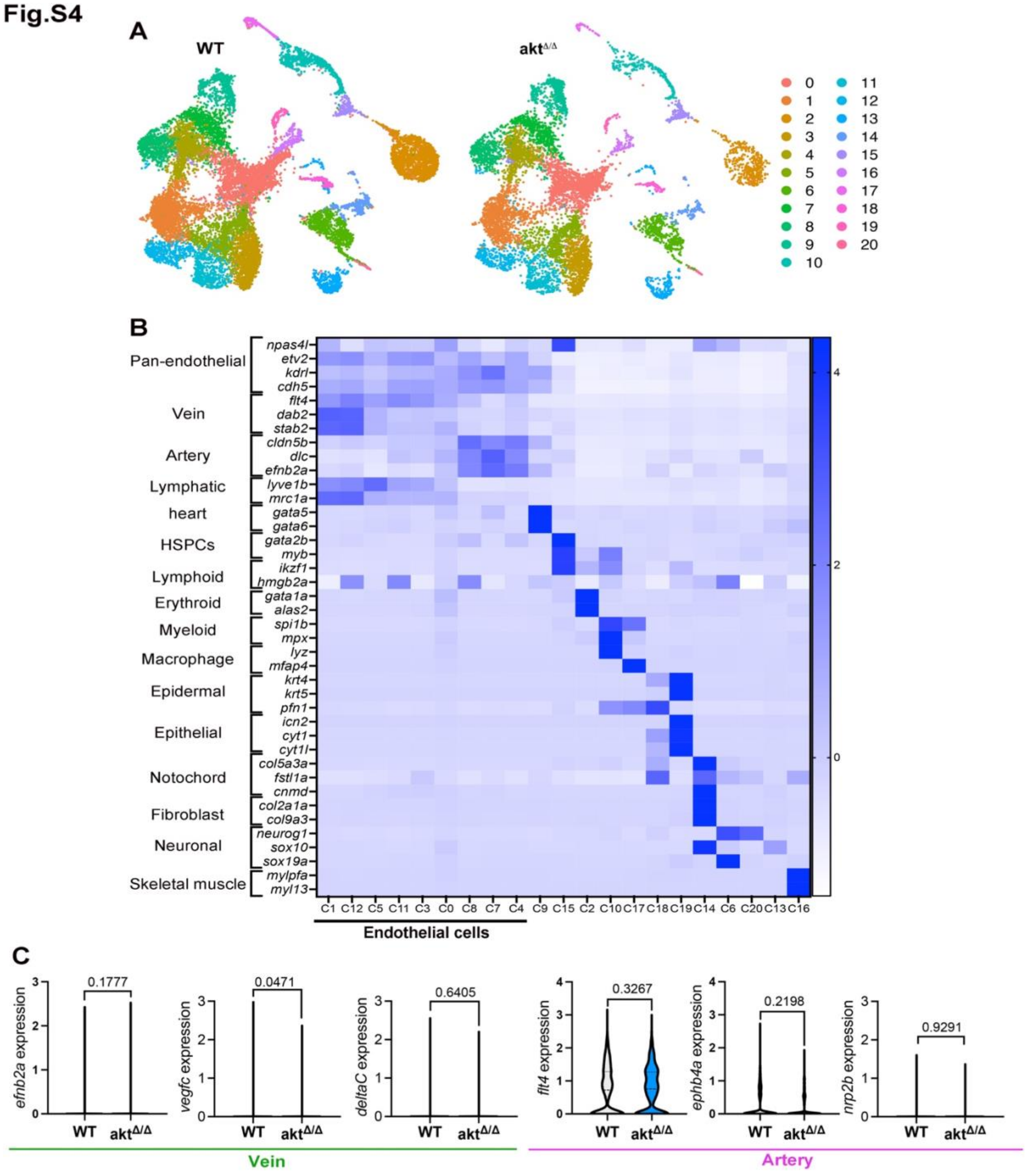

Fig. S4. Single-cell RNA sequencing strategy in WT and a kt$^{\Delta/\Delta}$.

(A) UMAP of all WT and akt$^{\Delta/\Delta}$ cells sequenced in our scRNAseq experiment. (B) Heatmap of gene marker expression corresponding to various cell types in all 21 cell clusters. Endothelial cells are part of clusters 1, 12, 5, 11, 3, 0, 8, 7 and 4. (C) Violin plot of *efnb2a, vegfc, deltaC; flt4, ephb4a* and nrp2b in arterial and venous clusters respectively (Mann–Whitney test). All quantifications are represented with mean ± s.e.m.

Development • Supplementary information

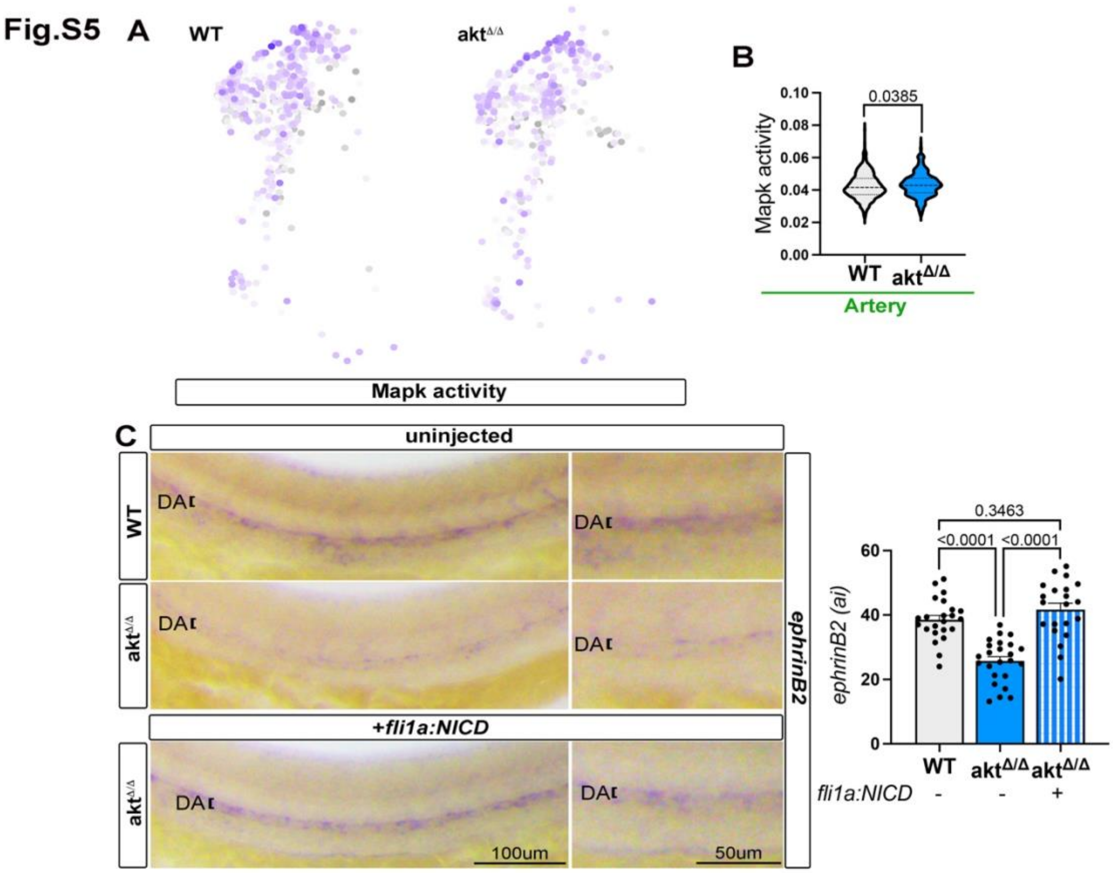

Fig. S5. Single-cell RNA sequencing strategy in WT and akt$^{\Delta/\Delta}$.

(A) UMAP of Mapk activity in WT and akt$^{\Delta/\Delta}$ artery cells. (B) Violin plot of Mapk activity in arterial cells (Mann–Whitney test). (C) *In-situ* hybridization against *ephrinB2* in WT and akt$^{\Delta/\Delta}$ injected or not with EC-specific NICD (+*fli1a*:*NICD*) at 24 hpf (n= 23 (WT), 24 (akt$^{\Delta/\Delta}$) and 21 (akt$^{\Delta/\Delta}$ +*fli1a*:*NICD*) embryos, ordinary one-way ANOVA). All quantifications are represented with mean ± s.e.m.

Development • Supplementary information

Figure S6
Raw western blots

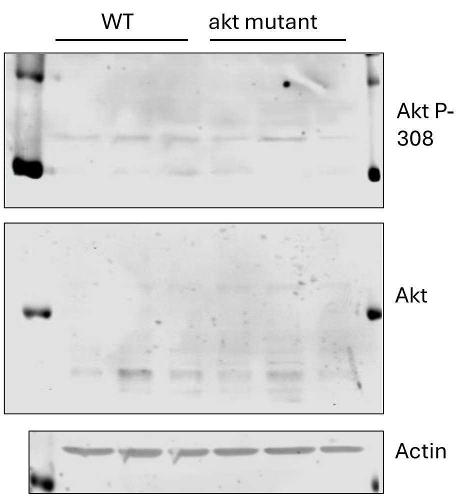

Raw western blot used in Figure 1D

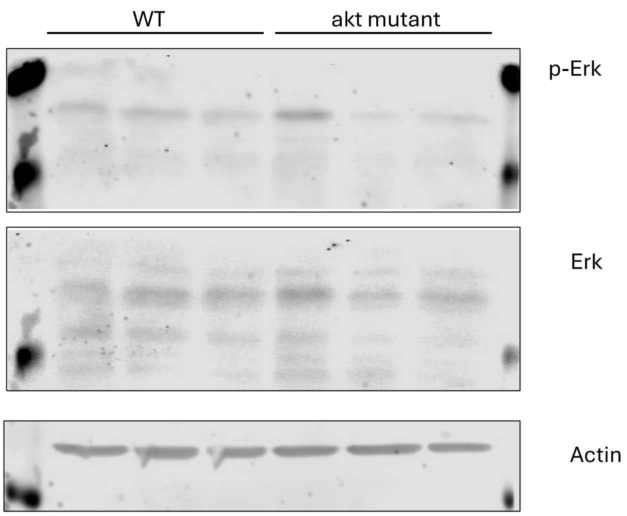

Raw western blot used in Figure S3A

Fig. S6. Raw images of western blots used in this study.

Development • Supplementary information

Table S1. gRNA, qPCR and genotyping primers used in the paper.

Primer name	F/R	Primer sequence (5'-3')	Assay
Akt1	F	TGCAGCGGTTAGGTGGAGGT	qPCR
	R	CTTGTCCAGGTGGTGTGATG	qPCR
Akt2	F	GATCCGCTTCCCTAGAAACC	qPCR
	R	GGTGGAACGAGCTTCTTTTG	qPCR
Akt3a	F	CAAGAGACTTGGTGGAGGTC	qPCR
	R	AGGAGAACTGGGGGAAGTGT	qPCR
Akt3b	F	CCAATAAGAGGCTCGGAGGA	qPCR
	R	CTGGCTTTCTCTCGCACCAG	qPCR
actin	F	TTCCTCCGCTTTCTCTGCAG	qPCR
	R	CCGAGCATCTCAGGGAATCC	qPCR
F-Akt1	F	AGGGCCTAAAGCTGATCCAT	genotyping
	R	CTGTGAGGCCACATTGCTAA	genotyping
F-Akt2	F	AGGAAGGGGTAGGGGTACAA	genotyping
	R	TGAGTCAAGGCACTCACCAC	genotyping
F-Akt3a	F	GGCCTGACGACTTAAGCAAA	genotyping
	R	CACTGGATTCGCTCCTCTTC	genotyping
F-Akt3b	F	GGGTCAAAAACAGGCCATAA	genotyping
	R	TGGGAGCGGTGATTAAATTG	genotyping
Akt1	F	GGGAAGGTGATTCTGGTGA	gRNA
Akt2	F	GGCATCCAGGCTGTGGCCAA	gRNA
Akt3a	F	GGCAGAGGCGATCCAGATGG	gRNA
Akt3b	F	GGCAGACAAGCTGGCCAAAC	gRNA

Table S2. Source data for all experiments part of this study.

Available for download at
https://journals.biologists.com/dev/article-lookup/doi/10.1242/dev.202727#supplementary-data

Development • Supplementary information

Table S3. Key resources table

REAGENT or RESOURCE	SOURCE	IDENTIFIER
Antibodies		
monoclonal rabbit phospho-Akt T308	cell signaling	Cat#2965; RRID: AB_2255933
monoclonal mouse pan-Akt	cell signaling	Cat#2920; RRID: AB_1147620
monoclonal mouse anti-actin	Sigma-Aldrich	Cat#A4700; RRID: AB_476730
chicken polyclonal anti-GFP	Abcam	Cat#ab13970; RRID: AB_300798
rabbit polyclonal RFP antibody	Antibodies- online	Cat#ABIN129578; RRID: AB_10781500
monoclonal rabbit phospho-ERK	cell signaling	Cat#4695; RRID: AB_390779
goat anti-rabbit Alexa Fluor 680	Thermo Fisher	Cat#A20984; RRID: AB_10375714
goat anti-chicken Alexa Fluor 488	Thermo Fisher	Cat#A11039; RRID: AB_2534096
goat anti-mouse Alexa Fluor 680	Thermo Fisher	Cat#A21057; RRID: AB_2535723
goat anti-mouse 800	Rockland	Cat#610-145-002-0.5; RRID: AB_11182794
Sheep anti-digoxigenin-AP-Fab	Roche	Cat#11093274910; RRID: AB_514497
Chemicals, Peptides, and Recombinant Proteins		
PTU (N-Phenylthiourea)	Sigms-Aldrich	Cat#P7629; CAS:103-85-5
NBT/BCIP	Roche	Cat#1681451
Complete Protease Inhibitor Cocktail	Roche	Cat#11 697 498 001
Pefabloc SC AEBSF	Roche	Cat#30827-99-7
PFA	Electron microscopy sciences	Cat#15710-S
Critical Commercial Assays		
ApopTag Red *in situ* Apoptosis Detection kit	Millipore Sigma	Cat#S7165
DC Protein Assay	Bio-rad	Cat#5000112
TOPO TA cloning kit	Invitrogen	Cat#K4500
iScript Reaction mix	Bio-rad	Cat#1708890
iQ SYBR green supermix	Bio-rad	Cat#1708882
Deposited Data		
Sc-RNA seq	This paper	GEO
Experimental Models: Organisms/Strains		
Zebrafish: wildtype AB	ZIRC	N/A
Zebrafish: akt1 ^{ya348}	This paper	N/A
Zebrafish: akt2 ^{ya349}	This paper	N/A
Zebrafish: akt3a ^{ya350}	This paper	N/A
Zebrafish: akt3b ^{ya351}	This paper	N/A
Zebrafish: *Tg(flt4: YFP)*^{hu4881}	N/A	ZDB-ALT-100208-1
Zebrafish: *Tg(kdrl: mCherry)*^{s916}	N/A	ZDB-ALT-090506-2
Recombinant DNA		
Tol2-Fli1a-NICD-v2a-mcherry	This paper	N/A
Tol2-Fli1a-mCherry-v2a-mcherry	This paper	N/A
Tol2-Fli1a-myr-Akt1-v2a-mcherry	This paper	N/A
Software and Algorithms		
Prism 9	Graphpad Software	https://www.graphpad.com/scientific-software/prism/
R Software	N/A	https://www.r-project.org/
Neurolucida	mbf Bioscience	https://www.mbfbioscience.com/neurolucida
Image J	NIH	https://imagej.nih.gov/ij/

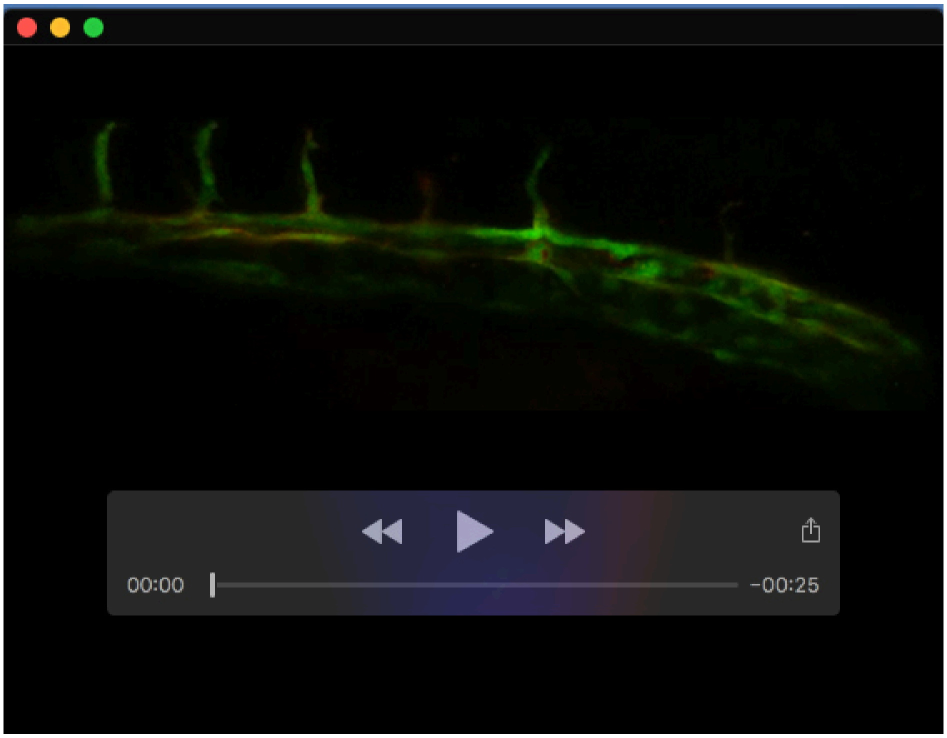

Movie 1. Time-lapse imaging of WT *(flt4:YFP; kdrl:HRAS-mCherry)*$^{hu4881;s896}$ embryos.

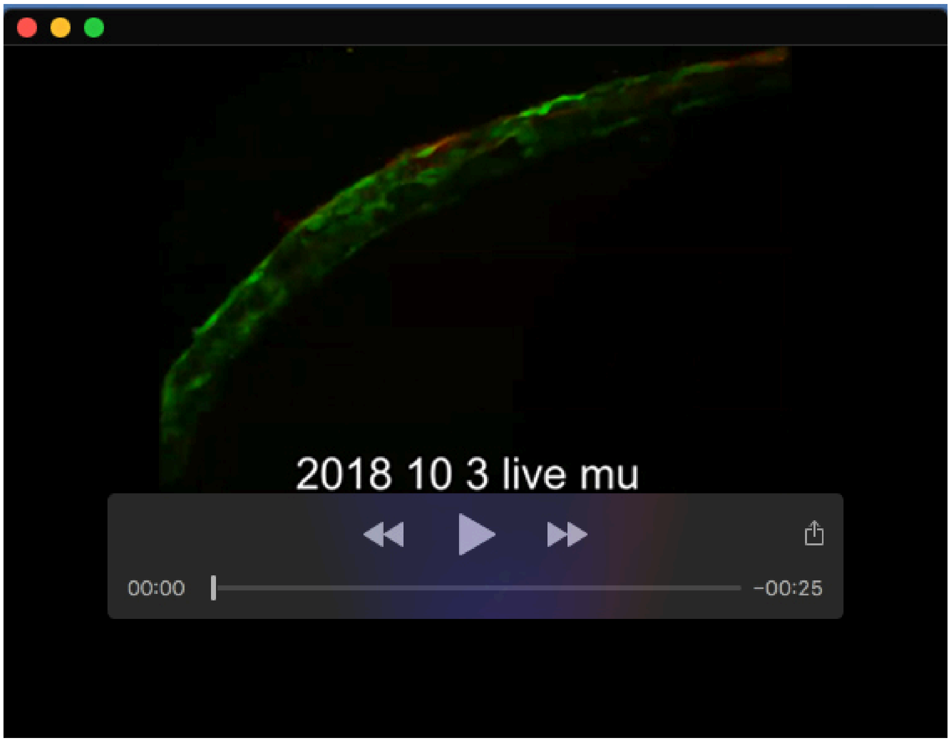

Movie 2. Time-lapse imaging of akt$^{\Delta/\Delta}$ *(flt4:YFP; kdrl:HRAS-mCherry)*$_{hu4881;s896}$ embryos.
