# Supplementary material for: Akt is a mediator of artery specification during zebrafish development
Source: Development. 2024 Sep 2;151(17):dev202727. doi: 10.1242/dev.202727 (PMC11441982; doi:10.1242/dev.202727)
Supplement: Supplementary file 1 [file develop-151-202727_review_history.pdf]

Akt is a mediator of artery specification during zebrafish development

Wenping Zhou, Joey J. Ghersi, Emma Ristori, Nicole Semanchik, Andrew Prendergast, Rong Zhang, Paola Carneiro, Gabriel Baldissera, William C. Sessa and Stefania Nicoli
DOI: 10.1242/dev.202727

Editor: Benoit Bruneau

Review timeline

Original submission:	23 January 2024
Editorial decision:	26 February 2024
First revision received:	21 May 2024
Editorial decision:	7 June 2024
Second revision received:	16 July 2024
Accepted:	16 July 2024

Original submission

First decision letter

MS ID#: DEVELOP/2024/202727

MS TITLE: Akt is a major mediator of artery specification during zebrafish development.

AUTHORS: Wenping Zhou, Joey J. Ghersi, Emma Ristori, Nicole Semanchik, Andrew Prendergast, Rong Zhang, Paola Carneiro, Gabriel Baldissera, William C. Sessa, and Stefania Nicoli

I have now received all the referees reports on the above manuscript, and have reached a decision. The referees' comments are appended below, or you can access them online: please go to BenchPress and click on the 'Manuscripts with Decisions' queue in the Author Area.

The overall evaluation is positive and we would like to publish a revised manuscript in Development, provided that the referees' comments can be satisfactorily addressed. Please attend to all of the reviewers' comments in your revised manuscript and detail them in your point-by-point response. If you do not agree with any of their criticisms or suggestions explain clearly why this is so. If it would be helpful, you are welcome to contact us to discuss your revision in greater detail. Please send us a point-by-point response indicating your plans for addressing the referees' comments, and we will look over this and provide further guidance.

Reviewer 1

Advance summary and potential significance to field

Zhou et al assess the role of the Akt family in zebrafish development. By deleting all 4 Akt genes, they find a specific role for this family in regulating the specification of the aorta. In particular, they find a decrease in arterial specification, while venous specification is unaffected. This is accompanied by a decrease in Notch signaling. This is a surprising role for PI3K/Akt signaling. The findings are novel and of substantial interest. The data supports the conclusions and is convincing. I have only a couple of suggested improvements.

© 2024. Published by The Company of Biologists under the terms of the Creative Commons Attribution License (https://creativecommons.org/licenses/by/4.0/).

Comments for the author

Major comments:
1) While the authors show that the major anatomical structures and expression of arterial-inducing factors are unaffected by Akt deletion, it is possible that the effect is not cell-autonomous. Cell transplantation experiments would be helpful to more conclusively demonstrate that Akt deletion in arterial progenitors leads to the specification defects.
2) Does induction of Notch signaling rescue aortic diameter? The authors show that expression of NICD increases deltaC expression, but what about other arterial specification markers and aortic diameter?

Minor comments:
1) Is pERK quantitatively different in Akt knock-outs?
2) The figures are not numbered, which makes it difficult to find particular figures.
3) The 'artery' and 'vein' labels are switched in Figure. S4C.

Reviewer 2

Advance summary and potential significance to field

The study by Zhou et al. examines the influence of Akt on artery specification in zebrafish embryos. In order to examine this question, the authors generate quadruple mutants in all akt genes present in zebrafish and analyze the vascular phenotypes of these mutants. They find direct connections between the dorsal aorta (DA) and the posterior cardinal vein (PCV), in addition to reduced DA and PCV diameters. Using single cell sequencing, the authors show that Notch signaling is specifically reduced in arterial endothelial cells (ECs) without an effect on venous EC transcription. Overexpression of Notch Intracellular Domain in ECs was able to rescue the expression of an arterial gene, dlc. Together, the experiments provide interesting insights into the function of Akt during vascular development, while showing surprisingly small defects. The data are presented clearly with appropriate controls and statistical analysis. Despite these merits, the study in its present form lacks focus and does not aim at investigating the mechanisms causing the vascular phenotypes the authors observe.

Comments for the author

1. The authors claim that they "analyzed the cellular and molecular mechanisms underlying artery hypoplasia…showing that akt is necessary for the proper specification of EC progenitors into arteries" (discussion). This claim is not supported by the data presented. The main phenotype of Akt mutants appears to be blood vessel hypoplasia (reduced diameters). The authors observe this in both the DA and PCV between 24 hpf and 48 hpf (Figure 2C, E). Apparently, at 5 dpf, the hypoplasia of the vein resolved, while DA hypoplasia is still apparent (Figure 1G). Despite their finding that both the DA and PCV are affected, the authors do not analyze the vein phenotype any further. They also do not investigate the cellular cause of DA hypoplasia. To determine whether similar cellular processes result in DA and PCV hypoplasia in akt mutants, the authors should investigate for example changes in EC numbers (proliferation) in both the DA and PCV. It appears that Akt influences both the artery and the vein in this respect. While it might be that downregulation of Notch signaling might cause artery hypoplasia (and vein hypoplasia is caused by a different mechanism), the simpler explanation would be that the same cellular mechanisms cause vein and artery hypoplasia and the artery in addition to this defect also lacks proper specification (which is a mild phenotype).

2. The authors focus on changes in arterial gene expression in their single cell sequencing data and stress that "the molecular programs that dictate the development of the DA and PCV are conserved across vertebrate models", page 3. This statement is not clear to me considering the data presented and the literature cited. The authors show that DA cells contribute to the PCV in early embryos (and cite a 2009 paper, Herbert et al.). Thus, arterial cells acquire a venous fate here. They then cite a paper by Hou from 2022 studying mouse embryos and state that at the early embryonic stage when they perform their single cell sequencing "widespread arterialization from venous cells is not fully complete". How do their time-lapse imaging and their single cell

© 2024. Published by The Company of Biologists under the terms of the Creative Commons Attribution License (https://creativecommons.org/licenses/by/4.0/).

sequencing data go together? Is the DA vein-derived or is the PCV DA-derived? Another publication in zebrafish described the conversion of arterial intersegmental vessels from an arterial into a venous fate (Quillien et al, Development 2014). It seems more that in zebrafish embryos early veins are derived from arteries, while in the mouse embryo early arteries are derived from veins, underscoring fundamental differences in DA and PCV development across vertebrate models. The authors need to explain these findings and amend the text accordingly.

3. The finding that Notch signaling is reduced in DA cells in akt mutants is interesting, but it is currently unclear whether this causes DA hypoplasia. Does overexpressing NICD also rescue DA diameters or only arterial gene expression?

4. The statement that single cell sequencing confirms lack of arterial specification (also reiterated in the discussion) is not convincing, as all EC populations are present in akt mutants (no lack of arterial ECs). The change in Notch signaling appears to be small and a DA can apparently form. The authors should tone down their statement that “akt is the key kinase upstream of the Notch-pathway that sustains artery specification” (discussion).

5. It is striking that ERK phosphorylation is not perturbed in akt mutant embryos with reduced Notch signaling. Previous studies showed that in embryos lacking Notch, ERK phosphorylation is higher in DA cells (Shin, 2016, Development). The authors should comment on this.

6. It is not clear, which ECs in the embryos injected with Fli:NICD express NICD. Transient DNA injections often produce embryos with mosaic expression of the injected DNA construct. The authors state that dlc expression in intersegmental vessels is unaffected by NICD expression and show an ISV that has sprouted from the DA and expresses dlc. However, it is not clear whether the ISV the authors show expresses NICD. The authors need to stain for NICD protein (I assume it is tagged) and show which ECs express NICD and how dlc expression is changed in these ECs.

7. No movies are provided that would show the ventral migration of DA cells (Figure 1). These movies should be included in the supplementary material.

First revision

<u>Author response to reviewers' comments</u>

Point-by-point response to reviewers.

We extend our gratitude to all reviewers for their thoughtful and valuable comments. Below, you will find our point-by-point responses to each reviewer's remarks highlighted in blue.

Reviewer 1 Advance summary and potential significance to field

Zhou et al assess the role of the Akt family in zebrafish development. By deleting all 4 Akt genes, they find a specific role for this family in regulating the specification of the aorta. In particular, they find a decrease in arterial specification, while venous specification is unaffected. This is accompanied by a decrease in Notch signaling. This is a surprising role for PI3K/Akt signaling. The findings are novel and of substantial interest. The data supports the conclusions and is convincing. I have only a couple of suggested improvements.
We thank the reviewer for the encouraging comments on the novelty and potential community interest. We appreciate the concerns raised and have addressed them with new data presented below.

Major comments:
1) While the authors show that the major anatomical structures and expression of arterial-inducing factors are unaffected by Akt deletion, it is possible that the effect is not cell-autonomous. Cell transplantation experiments would be helpful to more conclusively demonstrate that Akt deletion in arterial progenitors leads to the specification defects.

© 2024. Published by The Company of Biologists under the terms of the Creative Commons Attribution License (https://creativecommons.org/licenses/by/4.0/).

We appreciate the reviewer's constructive feedback. To address this, we have made revisions to the manuscript and conducted additional experiments. Since inducing loss of function in all four Akt alleles presents challenges in establishing causation, the phenotype associated with tissue development likely stems from the tissue-specific expression of one or multiple isoforms. Therefore, the Akt loss of function mutant serves as a novel model for examining how various tissues respond to the diminishment of Akt signaling. While our emphasis on vascular development serves as a proof-of-principle example, we acknowledge that investigating cell-autonomous functions will require more advanced genetic techniques such as tissue-specific knockouts.

Nevertheless, in direct response to this reviewer’s particular concern, we conducted new experiments to ascertain whether arterial specification, DA diameter, are linked to the loss of Akt-endothelial signaling:

Initially, expression of Notch Intracellular Domain (NICD) in fli1a+ endothelial cells, followed by VT2A mCherry, revealed that NICD-mCh expression in the DA alone is adequate to rescue both dlc and ephrinB2 artery marker expression (new Fig. 5C and S5C). Moreover, this intervention was effective in restoring arteriovenous connections, DA diameter, and PCV diameter (new Fig. 5D). These findings support the notion that reactivating downstream Akt-signaling in artery cells is ample to rescue other endothelial cell phenotypes, such as vein morphogenesis. Similarly, expression of a constitutively active form of Akt1 in fli1a+ endothelial cells led to integration into artery cells and normalization of DA morphogenesis, as well as PCV (new Fig. 5D).

Taken together, these results further validate the necessity of Akt signaling in artery cell specification and morphogenesis. While we attempted cell transplantations we encountered difficulties likely due to early defects in angioblast segregation impairing transplantation quality, we believe that these alternative results sufficiently address the reviewer's suggestion.

2) Does induction of Notch signaling rescue aortic diameter? The authors show that expression of NICD increases deltaC expression, but what about other arterial specification markers and aortic diameter?
The experiment and results requested by this reviewer are discussed in point 1 above.

Minor comments:
1) Is pERK quantitatively different in Akt knock-outs?
To quantitatively assess p-Erk, we conducted a western blot analysis at 24 hpf in both WT and akt mutant samples of both total Erk and p-Erk. Our findings revealed no discernible difference in either Erk or p-Erk levels, thus corroborating our assertion via immunofluorescence that p-Erk remains unaffected in akt loss of function mutants. This newly obtained result has been incorporated into new Figure S3A.

2) The figures are not numbered, which makes it difficult to find particular figures (Adapt figure development nomenclature).
We apologize to the reviewer for the lack of figures numbering. We added this on top of each figure.

3) The ‘artery’ and ‘vein’ labels are switched in Figure. S4C (Change figure).
We thank the reviewer for pointing out that. This has been modified and addressed these issues (new Fig. 4C-E and S4C).

Reviewer 2 Advance summary and potential significance to field
The study by Zhou et al. examines the influence of Akt on artery specification in zebrafish embryos. In order to examine this question, the authors generate quadruple mutants in all akt genes present in zebrafish and analyze the vascular phenotypes of these mutants. They find direct connections between the dorsal aorta (DA) and the posterior cardinal vein (PCV), in addition to reduced DA and PCV diameters. Using single cell sequencing, the authors show that Notch signaling is specifically reduced in arterial endothelial cells (ECs) without an effect on venous EC transcription. Overexpression of Notch Intracellular Domain in ECs was able to rescue the expression of an arterial gene, dlc. Together, the experiments provide interesting insights into the

© 2024. Published by The Company of Biologists under the terms of the Creative Commons Attribution License (https://creativecommons.org/licenses/by/4.0/).

function of Akt during vascular development, while showing surprisingly small defects. The data are presented clearly with appropriate controls and statistical analysis. Despite these merits, the study in its present form lacks focus and does not aim at investigating the mechanisms causing the vascular phenotypes the authors observe.
We thank the reviewer for the positive comments on the rigor of our analysis. We appreciate the concerns raised and have addressed some of them with new data.

Major comments:
1. The authors claim that they "analyzed the cellular and molecular mechanisms underlying artery hypoplasia…showing that akt is necessary for the proper specification of EC progenitors into arteries" (discussion). This claim is not supported by the data presented.
Overall, our manuscript demonstrates that the Akt loss of function mutant provides a novel model for investigating the responses of various tissues to the genetic reduction of Akt signaling. Although our focus on vascular development serves as a proof-of-principle example, exploring cell- and molecular-level mechanisms in cell-autonomous functions will necessitate more sophisticated genetic techniques, such as tissue-specific knockouts of the four alleles. Hence, we have rephrased this sentence to downplay some of the conclusions drawn in the discussion.

The main phenotype of Akt mutants appears to be blood vessel hypoplasia (reduced diameters). The authors observe this in both the DA and PCV between 24 hpf and 48 hpf (Figure 2C, E). Apparently, at 5 dpf, the hypoplasia of the vein resolved, while DA hypoplasia is still apparent (Figure 1G). Despite their finding that both the DA and PCV are affected, the authors do not analyze the vein phenotype any further.
We have conducted additional experiments demonstrating that expression of the Notch Intracellular Domain or constitutively active Akt1 specifically in artery cells (DA) is adequate to rescue the morphogenesis of PCV (new Fig. 5D). This suggests that the observed defect in veins morphogenesis during early development may stem from impaired arteriovenous dissociation caused by inappropriate expression of arterial genes. Furthermore, we speculate that the later defect in artery diameter may not only result from early impairment in artery morphogenesis but also from other abnormalities associated with vascular development, such as the recruitment of vascular mural cells. We have now addressed this in the discussion section.

They also do not investigate the cellular cause of DA hypoplasia. To determine whether similar cellular processes result in DA and PCV hypoplasia in akt mutants, the authors should investigate for example changes in EC numbers (proliferation) in both the DA and PCV. It appears that Akt influences both the artery and the vein in this respect. While it might be that downregulation of Notch signaling might cause artery hypoplasia (and vein hypoplasia is caused by a different mechanism), the simpler explanation would be that the same cellular mechanisms cause vein and artery hypoplasia and the artery in addition to this defect also lacks proper specification (which is a mild phenotype).
We conducted endothelial cell (EC) quantification in WT and akt mutants at 24-27 hpf, employing both counting EC nuclei in the trunk or quantifying ECs in different vessels via scRNA sequencing. Our analysis revealed that the number of ECs in akt mutants remains unchanged compared to WT in the DA, PCV, or ISV (refer to the figure 1 below and new Fig. S2B). Therefore, we cannot conclude that the reduction in vascular diameter is associated with a significant decrease in EC number. As a result, assertions regarding vascular hypoplasia have been mitigated.

Figure 1. The endothelial cell numbers are unaffected in akt mutants. (A) Quantification of number of endothelial cells in each vessels at 24 hpf from WT and akt mutant Tg(kdrl: mCherry; flt4:YFP) obtained during tunel assay (n= 5 WT and 5 akt mutant, Ordinary one- way ANOVA with Tukey's multiple comparison). (B) Proportion of veinous and arterial cells from our single cell RNA sequencing analysis. (C) Proportion of all cells part of the cluster identified as Vein or Artery after clustering.

2. The authors focus on changes in arterial gene expression in their single cell sequencing data and stress that "the molecular programs that dictate the development of the DA and PCV are conserved across vertebrate models", page 3. This statement is not clear to me considering the data presented and the literature cited. The authors show that DA cells contribute to the PCV in early embryos (and cite a 2009 paper, Herbert et al.). Thus, arterial cells acquire a venous fate here. They then cite a paper by Hou from 2022 studying mouse embryos and state that at the early

© 2024. Published by The Company of Biologists under the terms of the Creative Commons Attribution License (https://creativecommons.org/licenses/by/4.0/).

embryonic stage when they perform their single cell sequencing “widespread arterialization from venous cells is not fully complete”. How do their time-lapse imaging and their single cell sequencing data go together? Is the DA vein-derived or is the PCV DA-derived?
It's important to note the distinction between the time-lapse imaging, which focused on visualizing the morphogenesis of the DA and PCV in trunk vessels, and the scRNA sequencing, which involved EC isolation from whole embryos, including the cranial region. In the trunk the arterial vessels form first such as the DA and arterial ISV, while much of the artery plexus in the brain parenchyma for example forms after the establishment of venous plexus like the primordial hindbrain channel (Fujita et al., 2011). We appreciate the reviewer's assistance in rectifying this in the manuscript. Since the confusion we have removed the statement and the citation Hou 2022.

Another publication in zebrafish described the conversion of arterial intersegmental vessels from an arterial into a venous fate (Quillien et al, Development 2014). It seems more that in zebrafish embryos early veins are derived from arteries, while in the mouse embryo early arteries are derived from veins, underscoring fundamental differences in DA and PCV development across vertebrate models. The authors need to explain these findings and amend the text accordingly.
While we have made revisions to the manuscript text, it's worth noting that in mouse trunk’s development, arteries typically form before veins (Chong et al., 2011; Fish and Wythe, 2015), a pattern reminiscent of observations in the trunk region of zebrafish. Furthermore, numerous studies have utilized zebrafish as a discovery model for arteriovenous specification, underscoring its effectiveness for studying this topic (Geudens et al., 2019).

3. The finding that Notch signaling is reduced in DA cells in akt mutants is interesting, but it is currently unclear whether this causes DA hypoplasia. Does overexpressing NICD also rescue DA diameters or only arterial gene expression?
We appreciate the reviewer for bringing attention to this aspect. This is consistent with a similar comment noted from Reviewer 1. New experiments have been conducted and the results are described in reviewer 1’s point 1 section above.

4. The statement that single cell sequencing confirms lack of arterial specification (also reiterated in the discussion) is not convincing, as all EC populations are present in akt mutants (no lack of arterial ECs). The change in Notch signaling appears to be small and a DA can apparently form. The authors should tone down their statement that “akt is the key kinase upstream of the Notch-pathway that sustains artery specification” (discussion).
We express gratitude to the reviewer for the comment and acknowledge its significance. Consequently, we have revised the statement in the discussion on Page 16 and in the title to align with this reviewer's feedback. We have downplayed the role of Akt as upstream Notch’s regulator in artery differentiation.

5. It is striking that ERK phosphorylation is not perturbed in akt mutant embryos with reduced Notch signaling. Previous studies showed that in embryos lacking Notch, ERK phosphorylation is higher in DA cells (Shin, 2016, Development). The authors should comment on this.
We concur with the reviewer that this finding is particularly intriguing. Firstly, we would like to emphasize that in response to minor revision point 1 of Reviewer 1 (see above), we have quantified in 24 hpf embryo both total Erk and pErk via western blot analysis, leading to similar conclusions (new Fig. S3A).
Second, our results offer several potential explanations especially when considering its implications within the existing literature. In a study by Shin et al. (2016), it was demonstrated that embryos lacking notch activity (observed either in mindbomb mutants or through DAPT treatment) display a notable increase in p-Erk levels in the EC undergoing sprouting of the Intersegmental Vessels. This supports the notion that Erk is necessary for angiogenesis but dispensable for arterial specification (which is consistent with our observations). Furthermore, we would like to acknowledge that Shin et al’s experimental model entails a complete abolishment of Notch signaling, which contrasts with our experimental setup where Notch activity is only reduced, hence Erk activity might not be up-regulated adequately in the ISV to affect angiogenesis.

6. It is not clear, which ECs in the embryos injected with Fli:NICD express NICD. Transient DNA injections often produce embryos with mosaic expression of the injected DNA construct. The authors state that dlc expression in intersegmental vessels is unaffected by NICD expression and show an ISV that has sprouted from the DA and expresses dlc. However, it is not clear whether the

© 2024. Published by The Company of Biologists under the terms of the Creative Commons Attribution License (https://creativecommons.org/licenses/by/4.0/).

ISV the authors show expresses NICD. The authors need to stain for NICD protein (I assume it is tagged) and show which ECs express NICD and how dlc expression is changed in these ECs.
We appreciate the reviewer's suggestions. As correctly interpreted, NICD was tagged with a VT2A mCherry fluorescent protein to facilitate visualization of integrated NICD+ cells. We have included new images as well as quantification of the location and number of NICD-mCh+ cells. The integration of NICD-mCh was primarily observed in the DA's cells, thus we can primarily comment on the expression of artery markers there. Furthermore, it was noted that the expression of dlc in the Akt mutant ISV remains unaltered compared to the WT. Therefore, even if expressed in ISV cells, NICD shouldn't exert Akt-specific functions. However, the expression of artery markers is augmented in the Akt mutant's DA due to its specific expression and NICD downstream effects (see new Fig. 5C,D and S5C).

7. No movies are provided that would show the ventral migration of DA cells (Figure 1). These movies should be included in the supplementary material.
We apologize for the oversight. Both movies (WT and akt mutant) have now been uploaded in the revised version of the manuscript.

Reviewer 3, Advance summary and potential significance to field
In this manuscript, the authors generate zebrafish lacking the 4 genes encoding AKT homologs and characterize associated vascular development phenotypes. This approach is relevant and of interest to the vascular biology community as the role for AKT remains unclear in the context of early vascular development. The authors initially find late-stage lethality with decreased arterial diameters at late larval stages with AKT-deficiency. Early serial analysis suggested poor separation of artery and vein and in situ analysis suggested a decreased level of artery-specific gene expression. These observations were supported by scRNA-seq. The authors propose reduced Notch signaling as possibly leading to the observed phenotypes and attempt to demonstrate this through endotheial-specific activation of Notch in AKT-deficient embryos. While the results are suggestive, they are not particularly convincing and the experimental approach is lacking in a number of areas. Details are listed below.
We acknowledge this reviewer's feedback on our manuscript, which we are glad that it was considered of interest to the broader vascular biology community. We have incorporated additional experimental validations and revised the manuscript to address the limitations of our current experiments and results.

Major comments:
1. It appears that there is still protein from the Western blot in AKT mutants, but there is no explanation. It seems these were not genotyped based on the Methods. If this is the explanation, the authors need to more carefully use genotyped embryos to demonstrate there is no AKT protein made in the mutants. If there is remaining AKT protein, why is it there? Similarly, more rigorous assessment of downstream AKT effectors is important to document.
We would like to emphasize that confirmation of Akt loss of function was conducted using quantitative PCR on genotyped embryos (Fig. 1C). These results, along with the western blot data, indicated that 80-95% of the akt(s) mRNA was lost in the akt mutants, which aligns with the standard for acceptable loss of function mutant generated via premature stop codon gRNA/Cas9 models. Regarding the western blot analysis, we believe that demonstrating an 85% reduction in both total Akt and phospho-Akt provides sufficient evidence of Akt loss of the majority signaling properties. Since zebrafish lack specific antibodies recognizing the zebrafish Akt1,2,3a,3b proteins and their phosphorylated forms, it is reasonable to presume that the residual protein (less the 17%) could originate from the recognition of other kinases. For similarly reasons we can't address the lack of downstream Akt effectors via western blot analysis.

However, we have now conducted genetic manipulation of Notch- and Akt1-signaling in endothelial cells to partially restore arterial specification and morphogenesis through genetic expression vector experiments, supporting the reduction of Akt' downstream effectors in mutant's endothelial cells (see points below).

2. Arterial diameter is modestly reduced in AKT mutants. This could be an effect of flow, which is only checked via in situ for flow-responsive markers, which is not sufficient as there may be changes that do not elicit a strong molecular response detectable with this assay. The authors need

© 2024. Published by The Company of Biologists under the terms of the Creative Commons Attribution License (https://creativecommons.org/licenses/by/4.0/).

to quantitatively assess flow rates as well as blood cell numbers and heart rate. Quantification of mural cell coverage at 5 dpf is also important. To demonstrate endothelial autonomy, the authors need to perform the appropriate rescue experiment.

We conducted new experiments to ascertain whether arterial specification, DA and PCV diameter, are linked to the loss of Akt-endothelial signaling:

Initially, expression of Notch Intracellular Domain (NICD) in fli1a+ endothelial cells, followed by VT2A mCherry, revealed that NICD-mCh expression in the DA alone is adequate to rescue both dlc and ephrinB2 artery marker expression (new Fig. 5C and S5C). Moreover, this intervention was effective in restoring arteriovenous connections, DA diameter, and PCV diameter (new Fig. 5D). These findings support the notion that reactivating downstream Akt-signaling in artery cells is ample to rescue other endothelial cell phenotypes, such as vein morphogenesis. Similarly, expression of a constitutively active form of Akt1 in fli1a+ endothelial cells led to integration into artery cells and normalization of DA morphogenesis, as well as PCV (new Fig. 5D).

In response to the reviewer's request for additional phenotype clarification (e.g blood flow, mural cells number), we wish to clarify that our manuscript does not assert that all vascular defects in Akt-signaling impairment are solely attributable to endothelial cells. Indeed, we acknowledge that our manuscript cannot definitively establish the specific role of all Akt isoforms in vascular cells. There are four Akt isoforms to consider, which may be expressed in various vascular and non-vascular cell types. Therefore, elucidating the cell-autonomous role of Akt signaling at later developmental stage when the vascular system increased functional and cellular complexity would require generating four allele-specific endothelial cell knockout models, a task beyond the scope of our current study.

In summary, we have presented additional evidence highlighting the tissue-specific role of Akt in early artery specification. Furthermore, we have revised our manuscript to underscore the utility of our novel mutant as a valuable tool for screening developmental defects across entire tissues linked to genetic impairments in Akt signaling. As a demonstration of this utility, we prefer to highlight the example of artery specification.

3. The observed “failed to separate” phenotype is not particularly convincing. Better imaging would help. Also, it seems that DA and PCV completely separate at later stages based on the text. Does this mean that arteriovenous shunts don’t form? This is a hallmark defect associated with loss of artery identity. If shunts are not forming, then it is likely that modest loss of artery marker gene expression is not consequential here.
We present the discovery of arteriovenous connections in akt mutants as likely resulting from the loss of arterial specification. These connections can indeed be rescued by endothelial activation of NICD or Akt 1 (new Fig. 5D), indicating they are a direct consequence of Akt signaling loss in endothelial cells. Mutant Akt did not exhibit blood flow shunts, which typically result from the loss of both arterial and venous identity, as well as impaired blood flow response. Given this, we shouldn't anticipate the formation of blood flow shunts, as venous identity, proper blood flow gene expression, and circulation are maintained in akt-signaling mutants.

In essence, impairment of Akt signaling affects proper dorsal aorta specification without causing shunts. We deem this a significant phenotype.

4. The decreased artery gene expression is very modest and non-fluorescent in situ hybridization is not a quantitative approach. qRT-PCR from FACS-isolated cells is needed. Comparative assessment of similar cells from embryos lacking Vegfa or Notch would be helpful.
We have presented evidence of decreased artery gene expression using two distinct techniques widely accepted in the field, both yielding consistent results. Thus, our conclusions are bolstered by the coherence of these findings. Bright field in situ hybridization offers accurately quantifiable results using established quantification methods (Dobrzycki et al., 2020). This technique's advantage over qRT-PCR lies in its ability to spatially quantify artery gene expression directly in the dorsal aorta (DA), avoiding the averaging of expression across multiple vessels, which could otherwise obscure overall expression levels.

© 2024. Published by The Company of Biologists under the terms of the Creative Commons Attribution License (https://creativecommons.org/licenses/by/4.0/).

Similarly, in our single-cell RNA sequencing analysis, comparing artery marker expression solely within artery cells mirrors the approach of qRT-PCR analysis in FACS-sorted cells, as suggested by this reviewer. Taken together, we are confident that quantifying DA expression or artery markers supports our conclusions, and that our corroborating experiments sufficiently assess arterial marker expression.

5. From my reading, it seems that the scRNA-seq is only based on a single replicate? If so, the p-values calculated from expression differences are not really accurate – they would be considering number of cells, but not biological replicates.
We conducted single-cell RNA sequencing on a cohort of 500-1000 embryos per condition (WT or Akt mutant) hence multiple replicas were combined to achieve 10 thousands of endothelia cells for sequencing. Statistical analyses, including comparisons of cell numbers and expression levels (as depicted in violin plots), were performed by comparing the total number of cells (in the order of hundreds) in each condition, enabling rigorous statistical analysis. This methodology aligns with the practices adopted by numerous other publications in the field. We maintain that the nonparametric test, utilizing summation of ranks applied to independent samples, is the most appropriate statistical approach in this context.

6. “Notch activity” from scRNA-seq analysis is vague. The authors need to show relevant examples (particular genes) from the scRNA-seq data and then validate those by qRT-PCR. The authors should also use any of the well-known Notch reporters in zebrafish to validate their preliminary observation. If Notch activity is actually reduced, are there corresponding changes in cell numbers/proliferation/behaviors in the DA? If not, the observed changes would not be considered relevant in the context Notch’s canonical role in artery differentiation/angiogenesis.
Crossbreeding the Akt mutants with Notch reporters in zebrafish would dilute all four alleles, and reconstituting the full mutant offspring would take nearly one year. Although feasible, injecting Notch reporter plasmids may have limitations due to mosaic assessment of Notch activity, resulting in significant variability among embryos based on integration levels. Essentially, we don’t believe that this analysis will results in more rigorous conclusion, and we believe that our current data documenting the impairment of Notch-signaling is sufficiently documented at this stage:

Firstly, akin to qRT-PCR, single-cell RNA sequencing of FAC-sorted endothelial cells subsequently clustered into arteries revealed a statistically significant difference in specific artery markers but not venous ones in mutant cells. The statistical robustness is supported by the analysis of hundreds of cells per genotype.

Secondly, in addition to individual gene tests, we demonstrated a collective decrease in Notch signaling activity using a publicly available computational code that assesses the expression of the entire Notch-target gene set (Aibar et al., 2017). Therefore, the activity represents an average expression level of dozens of Notch target-genes.

Thirdly, we conducted whole-mount in situ hybridization of Notch-direct target gene, ephrinB2, alongside dlc, revealing a significant decrease in ephrinB2 expression in akt mutants, as predicted. Importantly, this decrease can be restored to wild-type levels in the dorsal aorta by specific expression of NICD in mutant cells, supporting the requirement of Notch-Activity in akt-mutnat cells to regulate ephrinB2 and dlc expression.

We wish to clarify that our manuscript does not propose Akt as a general regulator of Notch in all Notch-dependent functions. For instance, Akt is not required for intersegmental angiogenesis, which relies on Notch regulation. Therefore, Akt likely does not control Notch signaling in these vessels but primarily in the dorsal aorta. This observation is not unexpected, considering the intricate regulatory model of Notch. Additionally, it's important to note that other Notch functions outside of artery specification are beyond the scope of our current study.

7. The endothelial-specific rescue with N1ICD is not convincing. Transgene expression here would be mosaic and N1CD-expressing need to be co-visualized with the in situ. The criticism above regarding quantification of in situs stands. Furthermore, N1CD expression would prevent ISV sprout formation as previous papers have shown. Therefore, you might not expect an effect on ISVs with mosaic transgene expression as those cells would only be in the DA.

© 2024. Published by The Company of Biologists under the terms of the Creative Commons Attribution License (https://creativecommons.org/licenses/by/4.0/).

We appreciate the reviewer's suggestions. As correctly interpreted, NICD was tagged with a VT2A mCherry fluorescent protein to facilitate visualization of integrated NICD+ cells. We have included new images as well as quantification of the location and number of NICD-mCh+ cells. The integration of NICD-mCh was primarily observed in the DA's cells, thus we can primarily comment on the expression of artery markers there. Furthermore, it was noted that the expression of dlc in the Akt mutant ISV remains unaltered compared to the WT. Therefore, even if expressed in ISV cells, NICD shouldn't exert Akt-specific functions. However, the expression of artery markers is augmented in the Akt mutant's DA due to its specific expression and NICD downstream effects (new Fig. 5D).

Minor comments:

The figures lacked numbers.
We apologize for the absence of figure numbering. We have included them to facilitate the reviewer's understanding.

In many of the feature plots, visualization of expressing cells is hindered by non-expressing cells. Please use "order=TRUE" in FeaturePlot (or similar) to more accurately present the data.
We apologized for this issue. We fixed the UMAP for the Notch and Mapk signaling that were not clear due to lack or cell organization. Please find the new UMAP in Figure 5A,B and Supplementary 5A.

p6, second full paragraph – ". . .required for tissue-specific development. . .". There could be lots of reasons for the observed phenotypes, so I would suggest less strong language.
We edited this sentence accordingly to this suggestion at Page 8.

p9, first full paragraph – ". .48hpf when the circulatory system is fully formed. .". It is fully functional at this stage, but fully "formed" is probably not accurate (e.g. trunk ISVs are still remodeling).
We edited this sentence accordingly to this suggestion at Page10.

p10, second paragraph – ". .Akt-signaling is required for arteriovenous remodeling. ." This is not an accurate statement based on the presented data. "Arteriovenous remodeling" is not really what seems to be affected here.
We edited this sentence accordingly to this suggestion at Page 11.

References:
Aibar, S., González-Blas, C.B., Moerman, T., Huynh-Thu, V.A., Imrichova, H., Hulselmans, G., Rambow, F., Marine, J.C., Geurts, P., Aerts, J., et al. (2017). SCENIC: single-cell regulatory network inference and clustering. Nat Methods 14, 1083-1086. 10.1038/nmeth.4463.
Chong, D.C., Koo, Y., Xu, K., Fu, S., and Cleaver, O. (2011). Stepwise arteriovenous fate acquisition during mammalian vasculogenesis. Dev Dyn 240, 2153-2165. 10.1002/dvdy.22706.
Dobrzycki, T., Krecsmarik, M., and Monteiro, R. (2020). Genotyping and Quantification of In Situ Hybridization Staining in Zebrafish. J Vis Exp. 10.3791/59956.
Fish, J.E., and Wythe, J.D. (2015). The molecular regulation of arteriovenous specification and maintenance. Dev Dyn 244, 391-409. 10.1002/dvdy.24252.
Fujita, M., Cha, Y.R., Pham, V.N., Sakurai, A., Roman, B.L., Gutkind, J.S., and Weinstein, B.M. (2011). Assembly and patterning of the vascular network of the vertebrate hindbrain. Development 138, 1705-1715. 10.1242/dev.058776.
Geudens, I., Coxam, B., Alt, S., Gebala, V., Vion, A.C., Meier, K., Rosa, A., and Gerhardt, H. (2019). Artery-vein specification in the zebrafish trunk is pre-patterned by heterogeneous Notch activity and balanced by flow-mediated fine-tuning. Development 146. 10.1242/dev.181024.

Second decision letter

MS ID#: DEVELOP/2024/202727

© 2024. Published by The Company of Biologists under the terms of the Creative Commons Attribution License (https://creativecommons.org/licenses/by/4.0/).

MS TITLE: Akt is a mediator of artery specification during zebrafish development.

AUTHORS: Wenping Zhou, Joey J. Ghersi, Emma Ristori, Nicole Semanchik, Andrew Prendergast, Rong Zhang, Paola Carneiro, Gabriel Baldissera, William C. Sessa, and Stefania Nicoli

I have now received all the referees reports on the above manuscript, and have reached a decision. The referees' comments are appended below, or you can access them online: please go to BenchPress and click on the 'Manuscripts with Decisions' queue in the Author Area.

The overall evaluation is positive and we would like to publish a revised manuscript in Development, provided that the referees' comments can be satisfactorily addressed. Please attend to all of the reviewers' comments in your revised manuscript and detail them in your point-by-point response. If you do not agree with any of their criticisms or suggestions explain clearly why this is so. If it would be helpful, you are welcome to contact us to discuss your revision in greater detail. Please send us a point-by-point response indicating your plans for addressing the referees' comments, and we will look over this and provide further guidance.

Reviewer 1

Advance summary and potential significance to field

The authors present novel findings that demonstrate that Akt is involved in Notch signaling and artery specification by deleting all copies of the gene in zebrafish. This is a significant advance.

Comments for the author

The authors have thoroughly addressed the previous reviewer comments and have provided new data. The data largely support the conclusions. I have one suggested change in the abstract as I think the current statement is a bit too strong:

Suggest changing "The Akt-loss-of-function mutant unveils Akt kinase's position upstream of Notch, required for proper embryonic artery specification." to the following - "The Akt-loss-of-function mutant unveils that Akt kinase can act upstream of Notch in arterial endothelial cells, and is involved in proper embryonic artery specification." The authors do not demonstrate an absolute requirement for Akt in Notch signaling or artery specification, so the language should be toned down somewhat.

Reviewer 2

Advance summary and potential significance to field

The authors clarified some of my points, but others were inadequately addressed.

Comments for the author

Basically, they only added Figure panel 5D while all the other main figures remain identical to the previous version. Figure 5D is not clear to me. On the left of the images there is a “WT” box and a “akt delta-delta” box, while to the right of all the figure panels, there is a “akt delta delta” box spanning all image panels. Are the representative top images now from injected wt embryos and the bottom images from akt mutants? There should be at least 6 images here (injected wt and mutants for each of the constructs). The bigger problem is that the authors did not provide evidence that cells expressing mcherry (and therefore NICD in the NICD-mcherry injections) now cell autonomously show upregulation of arterial marker genes in akt mutants. This would be required to prove a cell autonomous function of akt signaling in arterial cells upstream of Notch signaling (as also requested by the other reviewers). The current image only shows that the respective transgene is being expressed.

© 2024. Published by The Company of Biologists under the terms of the Creative Commons Attribution License (https://creativecommons.org/licenses/by/4.0/).

Another problem here is that it is not clear what the authors compare their measurements to. Does overexpression of NICD increase DA diameters also in wt embryos? The % of diameter compared to wt embryos should be compared to wt embryos overexpressing NICD in a similar number of DA cells. Maybe NICD overexpression increases DA diameters by, let's say 10%, both in wt and in akt mutants? The actual diameter values (in um) would be needed here as well. It is not clear what the authors define as 100% DA or PCV diameter, as also for the wt diameters, the values are distributed around the 100% value. Thus, some wt embryos have 90% of diameter and others 110% of diameter. This is not clear.

I also do not understand the authors' statement that the NICD construct mainly integrates into DA cells. In the %integration graph NICD has similar percentage values for DA and PCV, while akt1 seems to integrate more into DA than PCV. Is there some mislabeling here? What does %integration refer to? Percent of cells in each embryo, percent of injected embryos? All of this is not clear.

There is no information in the methods section that would relate to figure panel 5D (injections, constructs, quantification).

The PDF with the response to reviewers did not show Figure 1 (The endothelial cell numbers are unaffected in akt mutants).

I cannot follow the authors' statement on page 9 "Consistent with the disrupted segregation of artery and vein cells, akt delta/delta mutants show a decrease of DA and PCV lumen formation compared to WT at 24hpf." As apparently (see above) EC numbers are not changed, how is it that both the artery and vein are smaller? A misdistribution of cells from the artery to the vein should lead to the artery containing more cells while the vein should have fewer cells. Thus, the vein should be smaller, and the artery should be bigger.

The new abstract reads weirdly compared to the previous version. Did an AI breathe life into it?

Second revision

Author response to reviewers' comments

Reviewer 1 Advance Summary and Potential Significance to Field:
The authors present novel findings that demonstrate that Akt is involved in Notch signaling and artery specification by deleting all copies of the gene in zebrafish. This is a significant advance.

Reviewer 1 Comments for the Author:
The authors have thoroughly addressed the previous reviewer comments and have provided new data. The data largely support the conclusions. I have one suggested change in the abstract as I think the current statement is a bit too strong: Suggest changing "The Akt-loss-of-function mutant unveils Akt kinase's position upstream of Notch, required for proper embryonic artery specification." to the following - "The Akt-loss-of-function mutant unveils that Akt kinase can act upstream of Notch in arterial endothelial cells, and is involved in proper embryonic artery specification." The authors do not demonstrate an absolute requirement for Akt in Notch signaling or artery specification, so the language should be toned down somewhat.

We thank the reviewer for this suggestion that is now included in the abstract.

Reviewer 2 Advance Summary and Potential Significance to Field:
The authors clarified some of my points, but others were inadequately addressed.

Reviewer 2 Comments for the Author:
Basically, they only added Figure panel 5D while all the other main figures remain identical to the previous version. Figure 5D is not clear to me. On the left of the images there is a "WT" box and a "akt delta-delta" box, while to the right of all the figure panels, there is a "akt delta delta" box

© 2024. Published by The Company of Biologists under the terms of the Creative Commons Attribution License (https://creativecommons.org/licenses/by/4.0/).

spanning all image panels. Are the representative top images now from injected wt embryos and the bottom images from akt mutants? There should be at least 6 images here (injected wt and mutants for each of the constructs).

We apologize for any confusion. We agree that the wild-type un-injected does not represent the proper control for this experiment. However, we included it to show a reference for how a normal DA and PCV diameter should appear when there is 100% diameter recovery in the case of full rescue. We now removed the un-injected WT. The control for this experiment is the DA diameters in Akt mutants injected with Fli1-mCH (negative control), compared to those injected with Fli1-NICD or Akt1(perturbation). The reported differences support the previous conclusion.

We also believe that WT injected with NICD or Akt1 would not serve as an appropriate control. It is known that overexpression of these major signaling pathways in a normal background can cause ectopic defects, making comparison with Akt mutants, which lack or have impaired signaling expression, unjustifiable. We speculate that ectopic NICD and Akt expression in Akt mutants, likely represent a reconstitution of normal Notch and Akt signaling (not a over- expression). This is supported by our analysis of artery marker expression in Akt mutants injected with Fli1:NICD versus WT, as shown in Figure 5C and Figure S5C.

The bigger problem is that the authors did not provide evidence that cells expressing mcherry (and therefore NICD in the NICD-mcherry injections) now cell autonomously show upregulation of arterial marker genes in akt mutants. This would be required to prove a cell autonomous function of akt signaling in arterial cells upstream of Notch signaling (as also requested by the other reviewers). The current image only shows that the respective transgene is being expressed.

We respectfully disagree with this statement. We provided the whole mount expression data for two artery markers, *delta c* and *ephrinb2*, in Fli1:NICD injected Akt mutants. Our results suggest that the expression of both artery markers in the DA increased with NICD overexpression. While the number of NICD-mCH positive cells is mosaic, the intensity of *delta c*+ and *ephrinb2*+ cells within the mosaic DA has significantly increased. Also, increased activity of Notch could affect Notch signaling in neighboring cells that could be Notch-NICD-mCH negative. We believe our data sufficiently supports our conclusion.

Another problem here is that it is not clear what the authors compare their measurements to. Does overexpression of NICD increase DA diameters also in wt embryos? The % of diameter compared to wt embryos should be compared to wt embryos overexpressing NICD in a similar number of DA cells. Maybe NICD overexpression increases DA diameters by, let's say 10%, both in wt and in akt mutants? The actual diameter values (in um) would be needed here as well. It is not clear what the authors define as 100% DA or PCV diameter, as also for the wt diameters, the values are distributed around the 100% value. Thus, some wt embryos have 90% of diameter and others 110% of diameter. This is not clear.

We agree with this reviewer's comment, which was addressed in the rebuttal to the point above. We have now reported the diameter value in micrometers (μm) as requested (new Figure 5D).

I also do not understand the authors' statement that the NICD construct mainly integrates into DA cells. In the %integration graph NICD has similar percentage values for DA and PCV, while akt1 seems to integrate more into DA than PCV. Is there some mislabeling here? What does %integration refer to? Percent of cells in each embryo, percent of injected embryos? All of this is not clear.

We apologize for this oversight. We inadvertently reversed the bar plot values, and we thank the reviewer for pointing this out. We have now provided a new graph with the correct column order (new Figure 5D)

There is no information in the methods section that would relate to figure panel 5D (injections, constructs, quantification).

We added information about panel 5D in the method section (called: "Plasmid expression constructs").

© 2024. Published by The Company of Biologists under the terms of the Creative Commons Attribution License (https://creativecommons.org/licenses/by/4.0/).

The PDF with the response to reviewers did not show Figure 1 (The endothelial cell numbers are unaffected in akt mutants).

We are not sure why the uploaded pdf file missed the Figure 1. We apologize for this, and reviewer can find the EC number analysis in Figure S2B.

I cannot follow the authors' statement on page 9 "Consistent with the disrupted segregation of artery and vein cells, akt delta/delta mutants show a decrease of DA and PCV lumen formation compared to WT at 24hpf." As apparently (see above) EC numbers are not changed, how is it that both the artery and vein are smaller? A misdistribution of cells from the artery to the vein should lead to the artery containing more cells while the vein should have fewer cells. Thus, the vein should be smaller, and the artery should be bigger.

Our statement suggests that artery and vein cells are not fully segregated into their respective vessels at the developmental stage when they normally are in wild-type (WT) embryos. Essentially, we observed that these cells are "stuck" between the vessels, not completely part of either the artery or the vein. Consequently, while we likely see the same number of cells as in WT, the diameters are reduced because these cells are not yet fully integrated into the venous or arterial walls, unlike in WT embryos.

The new abstract reads weirdly compared to the previous version. Did an AI breathe life into it?

The abstract has been rewritten to incorporate Reviewer 1' s suggestions.

Third decision letter

MS ID#: DEVELOP/2024/202727

MS TITLE: Akt is a mediator of artery specification during zebrafish development

AUTHORS: Wenping Zhou, Joey J. Ghersi, Emma Ristori, Nicole Semanchik, Andrew Prendergast, Rong Zhang, Paola Carneiro, Gabriel Baldissera, William C. Sessa, and Stefania Nicoli
ARTICLE TYPE: Research Article

I am happy to tell you that your manuscript has been accepted for publication in Development, pending our standard publication integrity checks.

© 2024. Published by The Company of Biologists under the terms of the Creative Commons Attribution License (https://creativecommons.org/licenses/by/4.0/).
